# A non-academic perspective on the future of lithium-based batteries

**DOI:** 10.1038/s41467-023-35933-2

**Published:** 2023-01-26

**Authors:** James T. Frith, Matthew J. Lacey, Ulderico Ulissi

**Affiliations:** 1 Volta Energy Technologies, 28365 Davis Pkwy, Warrenville, IL 60555 USA; 2 Scania CV AB, 151 87 Södertälje, Sweden; 3 Sphere Energy SAS, 250 Bis Boulevard Saint Germain, 75007 Paris, France

**Keywords:** Batteries, Energy, Energy storage, Materials for energy and catalysis, Engineering

## Abstract

In the field of lithium-based batteries, there is often a substantial divide between academic research and industrial market needs. This is in part driven by a lack of peer-reviewed publications from industry. Here we present a non-academic view on applied research in lithium-based batteries to sharpen the focus and help bridge the gap between academic and industrial research. We focus our discussion on key metrics and challenges to be considered when developing new technologies in this industry. We also explore the need to consider various performance aspects in unison when developing a new material/technology. Moreover, we also investigate the suitability of supply chains, sustainability of materials and the impact on system-level cost as factors that need to be accounted for when working on new technologies. With these considerations in mind, we then assess the latest developments in the lithium-based battery industry, providing our views on the challenges and prospects of various technologies.

## Abstract (web summary)

In the field of lithium-based batteries, there is often a divide between academic research and industrial needs. Here, the authors present a view on applied research to help bridge academia and industry, focusing on metrics and challenges to be considered for the development of practical batteries.

## Introduction

Lithium-ion batteries should be recognized as a “technological wonder”. From a commercial point of view, they are the go-to solution for many applications and are increasingly displacing lead-acid and nickel-metal hydride (NiMH) systems^1^. At the same time, they represent a prime example of the successful results of joint academic and industrial research.

**Fig. 1:**
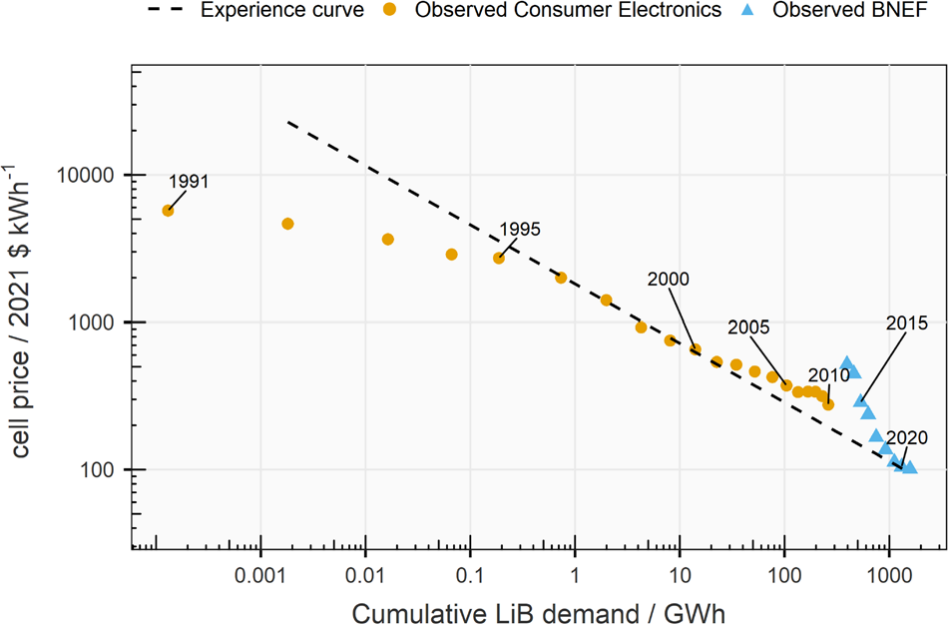
Observed lithium-ion battery cell prices 1991-2021. “Observed Consumer electronics” price data comes from ref. ^8^ and reflects the prices paid for cells used in consumer electronics between 1991 and 2010. “Observed BNEF” price data comes from ref. ^197^ and reflects the average price paid for cells used in electric vehicles and stationary storage applications. “Experience curve” shows the battery price decline trend as deployments increase. The relationship is described by Wrights-law and shows that every time the cumulative volume of cells deployed doubles, prices fall by 25%. Prices have been converted to real 2021 US $.

Lithium-ion batteries are complex, multi-component devices with a long list of inventors, key inventions, and contributions^2^. According to Akira Yoshino, lithium-ion batteries were born in 1986 after the successful safety testing of early prototypes^3^. Since then, the performance of lithium-ion cells (the fundamental building block of a battery pack) has improved substantially, and the specific energy and energy density have more than doubled from 120 Wh kg^−1^/264 Wh L^−1^ (Sony, 1991)^4^ to today’s ≥270 Wh kg^−1^/≥ 650 Wh L^−1^ ^5^. These values represent mass-produced commercial cells. Plants today typically produce over 1–10 GWh annually. Suppliers need to demonstrate the ability to manufacture at this scale to pass the stringent qualification tests of automakers and for the manufactured cells to be cost-competitive^6^. Mass production contributed to a sharp decline in cell prices, which fell 98% from *ca*. 5000 $ kWh^−1^ in 1991 to 101 $ kWh^−1^ in 2021 (Fig. 1)^7,8^. Low cost and high energy density cells resulted in the so-called “decade of the smartphone” around 2007^9^. Since then, demand for lithium-ion batteries has grown more than ten-fold, from ca. 30 GWh in 2011 to 492 GWh in 2021^10^. Demand is set to grow steadily and is forecasted to reach 2–3.5 TWh by 2030^11^. Growing demand for batteries can be expected to lead to further improvements in performance and falls in prices, with lithium-ion technology becoming ubiquitous.

Cost and performance improvements have come from cell chemistry/design changes, pack engineering, and manufacturing processes. Sony commercialized cells in 1991 using lithium cobalt oxide (LiCoO_2_ or LCO) “cathodes” and carbon-based “anodes”, in which the positive electrode active material is comprised of 60% cobalt by mass^12^. Note that from this point forward, we use “positive” and “negative” electrodes in place of the common terminology “cathode” and “anode” to avoid ambiguity since the latter terms are only valid for the discharge of a rechargeable battery.

The current state of the art^13^ lies in cells with specific energy over 270 Wh kg^−1^. These require a high nickel, low cobalt positive electrode active material, for example, lithium nickel manganese cobalt oxide (LiNi_1-a-b_Mn_a_Co_b_O_2_ where a + b = 1, or NMCxyz where x:y:z reflects the molar ratio of metals Ni:Mn:Co). A particularly important example is NMC811, which contains only 6% cobalt by mass. The low cobalt content means that the raw material cost, excluding processing costs (for example, raw material refinement cost or active/inactive material and cell manufacturing costs^14,15^), is less than half that of LCO: 54 $ kWh^−1^ compared to 135 $ kWh^−1^, based on January 2022 raw material prices from Shanghai Metals Market, SMM^16^. It is worth highlighting that these are spot prices, which may not be representative of long-term contract pricing.

Adopting new materials that increase energy content and decrease the raw material cost of cells has contributed significantly to reducing cell/pack costs ($ kWh^−1^). However, starting in 2020, similar improvements in both energy and cost have been obtained by employing existing positive electrode chemistries, such as lithium iron phosphate (LiFePO_4_ or LFP) in a cell-to-pack (CTP) configuration. In this configuration, an LFP-based cell with a specific energy of ca. 160 Wh kg^−1^ and energy density of 330 Wh L^−1^ can lead to pack-level energies of ca. 135 Wh kg^−1^ and 210 Wh L^−1^. This represents a 64% packing efficiency on a volume basis, compared to a 35–40% pack efficiency for a standard pack^17^. These CTP systems have the additional benefit of using a comparatively safer and potentially cheaper^18^ positive electrode active material than NMCxyz.

As lithium-ion batteries and the current generation of positive electrodes, i.e., those based on intercalation reactions, are reaching their theoretical performance limits, manufacturers and researchers are focusing on other key components and processing techniques. Negative electrodes with high silicon content, lithium metal negative electrodes, solid electrolytes, negative electrode pre-lithiation strategies and dry electrode coatings promise decreased cost, increased performance or both in the medium term (5–10 years). Looking further out, positive electrode active materials based on conversion reactions, like sulfur or oxygen, could present an opportunity for the further cost reduction of lithium-based batteries, although generally at the expense of cell performance.

However, particular attention must be devoted to the type of research carried out to advance lithium-based batteries. Indeed, as also recently discussed^19^, researchers should consider the current trajectory of battery technology, how to approach the industry and to present their work to provide the maximum benefit to the research community.

When carrying out research focusing on industrial product development, researchers should develop products that solve a problem rather than develop a solution that needs to find a problem to solve. We believe that lithium-ion batteries are an example of an industrial product, and research should focus on solving existing problems with the technology. However, a growing portion of research published on lithium-based batteries today does little to solve the industry’s challenges. Often this result from a lack of understanding of the wider end uses and performance parameters required for lithium-based batteries in end applications.

In this perspective, we present a non-academic view on applied research in electrochemical energy storage to help bridge the gap between academic and industrial research. We primarily consider lithium-based batteries, focusing on the automotive sector: a sector that has driven technological development in recent years, dominates today’s demand and is expected to grow significantly in the coming years. While we recognize that there are other emerging technologies, such as Na-ion batteries, as well as other application sectors, such as stationary energy storage, we choose to focus on electric vehicles (EVs), which are a core area of the energy transition. However, we recognize that these other topics warrant their separate discussions. To illustrate this perspective, we discuss technology maturity scales and what we believe are common pitfalls when evaluating performance requirements to bring a technology to market. We then select a few technologies as case studies. We use these to discuss what we believe the market will need and not need, provide practical, numerical examples, consider opportunities and barriers when scaling up, and ultimately explore which technologies currently show distinct promise.

## Discussion

### Technology readiness level from the lithium-ion battery perspective

**Fig. 2:**
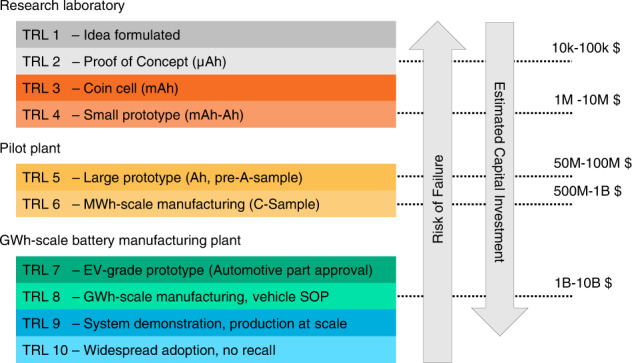
Technology readiness level scale for EV battery application. The “Risk of Failure” arrow indicates risks of project failure or technology not transitioning to the next level. The scale starts with lab innovation and considers key milestones in cell manufacturing to reach EV qualification and vehicle Start-of-Production (SOP). The definitions of A- and C-samples are discussed later in the “Challenges in scaling up” paragraph. Risk increases with decreasing TRL number. US dollar figures are ballpark estimates of the minimum investment required per project based on industrial data or publicly available press releases. The present TRL scale is based on the consideration of energy storage innovation disclosed in ref. ^198^.

First proposed by NASA in 1974, the Technology Readiness Level (TRL)^20^ is a scale used to estimate the maturity of a technology. Although a specific TRL scale has been recently proposed for battery manufacturing^20^, in Fig. 2, we propose a different TRL scale that considers the steps required for EV adoption to help decision-makers assess the actual status of technology development on the pathway to commercialization.

Technologies at a lower TRL are associated with a higher risk of project failure or technology not transitioning to the next level. However, this risk is offset by lower capital investments required to complete a project, e.g., 10k-100k $ at TRL 1-2 for battery science. Moving across TRLs generally requires increasing levels of capital investments. For example, over 1–10B US $ are the typical investments required to scale-up battery cell production to 4–20 GWh annually and reach vehicle Start-of-Production (SOP) at TRL 8 or to develop a new EV platform/powertrain and manufacture a vehicle at scale TRL 9-10. The capital figures are ballpark estimates of the minimum investment required per project based on industrial data or publicly available press releases.

Academic researchers usually operate at TRL 1–4, so they are generally less concerned with or unexposed to end-user requirements or criticalities that need to be considered when scaling up and manufacturing an energy storage device. Batteries in a research laboratory are often tested using conditions and parameters very far from commercial devices^21^. Moreover, scientific research in electrochemical energy storage is generally plagued by misrepresentation of data and a lack of transparency. This leads to a high risk of over-extrapolation, exacerbated by a lack of reproduced or even reproducible studies. Criticism of this situation is often kept within the community but has recently been spotlighted by various commentary and editorial articles^22–24^.

Within the battery industry, there have been several high-profile examples of companies investing in over-hyped technologies which failed to meet the promised performance. For example, Envia, a spin-out from Argonne National Laboratories (USA), was close to securing an investment from automaker General Motors to bring the technology to mass market EVs. However, the latter could not reproduce the results that Envia claimed, eventually leading to the demise of Envia^25^. Similarly, in 2015 the consumer products company Dyson acquired the US-based solid-state battery start-up Sakti3 for 90 million US $. Three years later, in 2018, the company wrote off the investment^26^.

### Practical evaluation of lithium-ion battery performance

Battery research and development is strongly driven and judged on a series of metrics with an often-complex connection between the requirements set by the application and the cell itself. For an EV, requirements on safety, range, available pack installation space, cost, power, and lifespan will heavily inform requirements at the cell level, such as energy density, chemistry, cell design, as well as calendar and cycle life. These requirements will depend not only on the demands of a specific application but also on other factors, such as legally mandated safety requirements in target markets.

Research into new battery chemistries (e.g., lithium-sulfur, solid-state, sodium-ion) and other concepts (e.g., redox flow, metal-air), regardless of application, has for many years been heavily driven by improving on these metrics, particularly (but not limited to) energy density, cycle life and cost. These metrics have a complex relationship between the material properties typically investigated at the fundamental research stage and the eventual application. We can take energy content on a weight or volume basis as a relevant example.

**Fig. 3:**
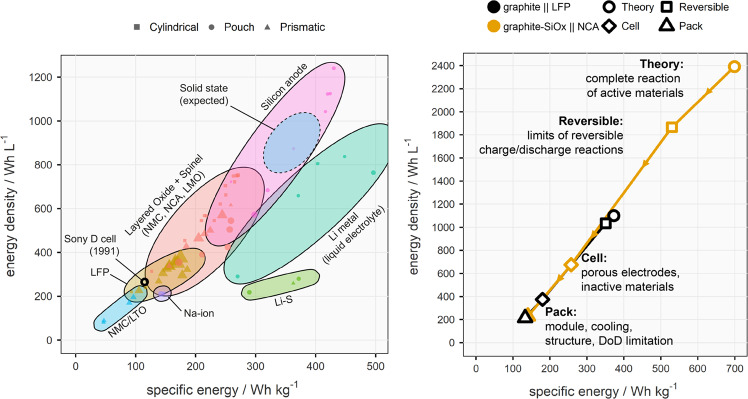
Visual representation of the range of energy content of different battery technologies at the cell level and energy losses between theory and system level. Left) energy density vs specific energy for selected Li-ion and “post-Li-ion” cells from publicly available specifications; right) schematic of the reduction in energy on a weight and volume basis between the theoretical maximum for the active materials and usable pack-level energy density for state-of-the-art NCA and LFP battery technologies. The symbols on the left chart are scaled based on cell size in terms of Ah. The data on which this figure is based are reported in Supplementary Note 1. Error bars are smaller than the data points for Fig. 3 right, and the reader is referred to Supplementary Note 1 for the range of values used. “DoD” refers to “depth of discharge”, the utilized fraction of the battery’s nominal capacity.

The left panel of Fig. 3 presents the specific energy (Wh kg^−1^) and energy density (Wh L^−1^) for a broad selection of Li-ion and so-called “post-Li-ion” cells^27^ with publicly available specifications grouped by chemistry type. A list of cell specifications used to construct this plot is given in Supplementary Table 1. Commercially available Li-ion batteries range from as low as ~50 Wh kg^−1^, 80 Wh L^−1^ for high-power cells with a lithium titanium oxide (Li_4_Ti_5_O_12_ or LTO) negative electrode, up to around ≥270 Wh kg^−1^, ≥650 Wh L^−1^ for cells with high-energy layered oxide positive electrodes (e.g., NMC811) and blended graphite/silicon composite negative electrodes^28^. Various prototypes of battery technologies under development, particularly those with pure silicon or lithium metal negative electrodes, show encouraging results in the development of high-energy cells^28^. However, graphical representations such as the left panel of Fig. 3 do not always allow us to understand the practical hurdles to translating single-cell performance into expected system-level performance. Moreover, these graphs do not necessarily predict where new battery chemistries may fall.

A schematic depiction of this in the context of energy is given in the right panel of Fig. 3, which describes the reduction in specific energy (Wh kg^−1^) and energy density (Wh L^−1^) from the theoretical level (“Theory”, which considers the calculable maximum energy release of the electrochemical reaction of the fully charged active materials, assuming no other inactive component) to the installed device (“Pack”, which considers structural and auxiliary components, among other practical limitations). This comparison is based on two contrasting state-of-the-art battery pack concepts: one based on small, high-energy-density cylindrical lithium nickel-cobalt-aluminium oxide (NCA) or high-nickel NMCxyz, in 18650 or 2170 cylindrical format cells, as currently used by companies such as Tesla. The other is based on large format LFP cells, such as those used in CTP concepts developed by companies such as BYD (“Build Your Dreams Co. Ltd.”) and CATL (“Contemporary Amperex Technology Co. Limited”) in which packing efficiency is increased by eliminating the use of smaller modules within the pack. The calculations carried out to produce the graph in the right panel of Fig. 3 are disclosed in Supplementary Note 1. The numbers should be interpreted as guidelines for these specific examples to highlight the crucial differences and not as descriptions of the full range of possible systems.

These two pack concepts contrast significantly at each stage of their implementation. From the right panel of Fig. 3, it can be seen that NCA (with a small, e.g., 3.5 wt. % inclusion of silicon oxide in the negative electrode) has approximately double the theoretical energy density of the graphite||LFP chemistry due to a higher cell voltage, capacity, and material density. However, the fraction of the theoretical energy content that can be reversibly obtained (repeatedly charged and discharged) is presently smaller for graphite-SiO_x_||NCA than graphite||LFP. Constructing a functioning rechargeable Li-ion cell requires the addition of inactive weight and volume, such as current collectors, separators, electrolyte, and packaging, which can be 50% by weight or more of the cell and reduces the energy density accordingly. For large systems such as EV batteries, comprising hundreds or thousands of cells, the cells must be installed into a pack with additional structural components and auxiliary systems such as cooling and electronic control. Other practical limitations might be required to realize certain requirements. For example, packs based on high-Ni-content NMC or NCA chemistries are typically limited further in terms of charging voltage (i.e., state-of-charge (SoC) and depth-of-discharge (DoD) ranges) to ensure an acceptable lifetime; the same limitations do not bind LFP-based batteries.

System (pack)-level design considerations may differ considerably with different chemistries; we can consider the comparison in the right panel of Fig. 3 as an example. Small, high-energy density cylindrical cells using high nickel content positive electrodes, with <20 Wh stored energy, are preferred by some original equipment manufacturers (OEMs) as thermal propagation in the event of thermal runaway can be more easily managed. Here we consider OEMs to be companies that produce battery packs. Other companies can use these packs as components to produce finished items, such as EVs, sold to users.

In contrast, the good thermal stability of LFP allows for relatively large (300–1000 Wh) cells with lower energy density and less stringent thermal management requirements. This fact, coupled with innovation in cell design, has recently enabled the development of LFP packs with improved packing efficiency, enabling pack-level energy densities competitive with high-Ni-content packs with energy-dense cells. However, recent announcements by several companies on innovations such as larger-format cylindrical cells (e.g., “4680”)^29^ and NMC-based CTP systems^30^, as well as further integration (e.g., cell-to-vehicle concepts, where the pack forms part of the vehicle structure)^31,32^ show that we can expect significant advancements in system-level engineering in the coming years, hence increased “cell-to-pack efficiency” (i.e., cell energy divided by pack energy, either gravimetric or volumetric) for NMC/NCA-based battery systems.

Figure 3 also implies that lithium-ion cells have been continuously optimized. Achieving today’s cell performance has been far from trivial, requiring a holistic approach to research and development and three decades of incremental improvements since market introduction. Because the positive electrode active material provides energy to the system during discharge, ideally, the mass and volume of all other components should be minimized while maximizing cell lifetime and performance without compromising safety. To achieve these targets, it is essential to realistically acknowledge the state-of-the-art and what are, or could be, practical constraints when conceiving a design of experiments. One should consider key variables, often referred to as key performance indicators (KPIs), such as the ratio of the capacities of the negative relative to the positive electrode (“N/P ratio”), practical electrode capacities, coating thicknesses, porosities and electrolyte loadings (Table 1). Typical lab-scale cells generally fall short of this in many respects: routine experiments use a large excess of Li metal and electrolyte. These factors can readily mask practical performance and lifetime achievable at both cell and system levels.

**Table 1:** Comparison of a few KPIs for a 2032 lab scale Li metal coin cell (1-10 mAh) with a commercial lithium-ion cell used in a Volkswagen ID.3 electric car^195^

KPI	Lab-scale Li metal coin cell (1-10 mAh)	Automotive Li-ion pouch cell (78 Ah)
Pos. el. areal capacity (mAh cm^−2^)	«2	5.02
Neg. el. areal capacity (mAh cm^−2^)	»20	5.23
N/P ratio	>10	1.04
Electrolyte loading (g Ah^−1^)	»30	1
Pos. el. coating thickness (µm)	<60 µm	87.3
Pos. el. porosity	»40%	22%

The numbers for the 2032 coin cell are reported as an example, and similar values are discussed in the literature^19^. For this example, we consider a coin cell with a discharge capacity of up to 4 mAh, uncalendered electrodes’ with a diameter of 1.6 cm, 100 µm thick lithium foil, and 100 µL of electrolyte. The greater/less-than symbols are used to stress that several examples of experiments deviate even more from these values. Values such as electrode coating thickness and porosity are not often reported, but constitute a key metric that can mask battery rate capability and lifetime^19^. The values reported in the table are for a single automotive cell. Values can vary depending on teardown methodology and actual cell design^105,196^.

#### The risk of excessive extrapolation

Over-extrapolation of early findings in battery research and development presents risks to the appropriate direction of public and private funding and policy decisions. In this context, over-extrapolation may often be fallacious inferences of future performance related to new materials beyond the experiments’ scope. For example, from results obtained in prototypical laboratory coin cells using Li metal as a counter/reference electrode^33^, a nanostructured positive electrode might indicate the possibility of batteries that fully charge in seconds, or a new negative electrode material might indicate better than state-of-the-art capacity retention. Such lab-scale cells are often free of several limitations that govern practical applications^21^. Over-extrapolation of this sort may be made by journalists^34^, by university press offices^35^, and, in some cases, by scientists authoring peer-reviewed scientific articles due to the often extreme pressure to motivate research funding.

A prominent recent example of excessive extrapolation is the 2016 *Energy & Environmental Science* research article by Braga et al.^36^ of a battery concept in which the alkali metal (Li or Na) was stated to reversibly plate and strip at both negative and positive electrodes with an extremely high theoretical energy density, despite the absence of an overall chemical reaction. The study gained worldwide attention following a university press release^37^. However, the study also received strong criticism and was subsequently disputed on theoretical and experimental basis^38,39^. At the time of writing, the peer-reviewed results obtained by Braga et al.^36^ have not been independently reproduced, and the papers disputing their results have received far less attention.

It is critical to scientific integrity and appropriate use of public resources that research funding organizations do not incentivize over-extrapolation at any level and support initiatives to improve data availability and transparency. In this regard, since 2015^40^, various scientific publishers and journals have suggested the development of standards in reporting experimental results and analysis in the broader field of energy research^24,41–46^. Another practice to support reproducibility and third-party validation is the publication of raw datasets. Indeed, the creation of community-led, open databases has already been considered in the battery field^47,48^. Another option could be to encourage the adoption of a “limitations of the study” section in peer-reviewed scientific articles as a standard practice, similar to that applied in other fields, notably the social sciences^49,50^. In this way, the scientists can clearly discuss methodological limitations, and the authors can clarify what remains outside the scope of their study in the article itself.

### Industrial development of lithium-based battery components

#### Electrolytes

A. Volta^51^ first described the importance of the electrolyte (i.e., an electron-insulating and ion-conductive layer, either liquid or solid, interposed between the negative and positive electrodes) in an electrochemical energy storage device in 1800. Currently, the state-of-the-art electrolyte for EV application^52–54^ is represented by solid lithium salts, e.g., lithium hexafluorophosphate, dissolved in non-aqueous organic-based carbonate solvents, e.g., ethylene carbonate and dimethyl carbonate. Electrolytes generally represent, depending on cell format and design, ca. 8–15 wt. % of a cell. Despite being continuously developed, these electrolytes are expected to continue limiting cell safety due to their combustibility and limited cell operating temperature range of −10 °C to 60 °C in the most optimistic scenarios.

Electrolyte chemistry plays a major role in determining cell safety, cycle life^55^, power capability, and reversibly accessible energy content^55,56^. It plays a key role in determining the nature of the so-called solid electrolyte interphase (SEI) forming at the interface between the electrolyte and the active material, especially at the negative electrode^57,58^. For most commercial battery cells, these kinetically stable interphases are critical for preventing the cell’s capacity and power degradation.

Moreover, innovative electrolyte formulations are considered key enablers for next-generation negative (e.g., lithium metal^59^ and silicon^60^) and positive (e.g., Mn-rich and polyanionic compounds^61^) electrode active materials. Academic and industrial researchers are trying to develop tailored liquid electrolyte formulations, e.g., using fluorinated solvents^62^ to enable efficient lithium metal cycling^59,63^. Room-temperature ionic liquids (RTIL, i.e., a class of salts that are liquid at room temperature) are also being considered^53,64^. Although RTILs are often touted as being safer than standard non-aqueous carbonate-based electrolytes^53,64^, there is limited evidence of long-term stability at TRL ≥ 5, particularly after an extended number of cycles. Start-up Cuberg has recently shown a cycle life of more than 670 cycles for a 5 Ah cell prototype containing an IL-based electrolyte^65^.

There is a strong push from the automotive industry to consider organic or inorganic solid-state electrolytes and so-called “solid-state batteries” (SSB), arguably among the most hyped technologies of this decade so far^66^. Unfortunately, despite the large volume of work reported in the scientific literature^67–69^, no consistent and comprehensive classification is available for all-solid-state batteries. For this reason, in Supplementary Fig. 1, we propose a classification to help guide the readers in what is being actively researched in the field.

We identify two main categories of all-solid-state cells: (i) thin film, with capacities in the µAh-mAh (or µWh-mWh) range which are already commercially available^70,71^, for example, in medical devices, smart electronics and circuit boards. These thin film batteries are generally produced by vacuum/vapour deposition, a technique which generally leads to low cell manufacturing throughput, compared to cell manufacturing for EV traction batteries^72^, and (ii) bulk-type, which are comparable, in principle, to current generation commercial lithium-ion batteries, i.e., with thick electrodes (~100 µm) and sizes ranging between 2 and 200 Ah. Below we summarise the various material approaches to solid-state electrolytes.

##### Inorganic solid-state electrolytes

Inorganic solid-state electrolytes are already available in niche commercial electrochemical energy storage devices such as high-temperature rechargeable, liquid electrode Na-S, Na-NiCl_2_ batteries used for stationary energy storage^73^ and primary Li-I_2_ batteries^73^. More recently, in 2019, Hitachi Zosen, a Japanese engineering corporation, showcased an all-solid-state 140 mAh pouch cell prototype for space-based applications that will be trialled on the International Space Station (ISS)^74,75^. The Hitachi Zosen cell uses a sulphide-based electrolyte with other undisclosed cell components and operates between −40 and 100 °C^74,75^, retaining performance at environmental pressures of 0.01 Pa^74,75^. Although this could be an advanced prototype in aerospace, sitting at least at TRL 7 for this niche application, it would sit at TRL 4 (i.e., laboratory scale) for EV application. Unfortunately, as of today, there is no off-the-shelf product that meets the stringent requirements of the passenger electric vehicle market.

Nevertheless, some solid-state electrolyte technologies hold much promise. For example, some inorganic solid electrolytes are stable and retain high ionic conductivities at room temperature^76,77^, e.g., > 10^−2^ S cm^−1^, while at the same time possibly improving safety due to a lower risk of thermal events^78^. These advantages could lead to increased volumetric and gravimetric energy at the pack level, i.e., by reducing the need for thermal management or engineering safety components around the battery pack.

The different nature of the electrode|solid electrolyte interface might also enable long-term cycling of negative (e.g., lithium metal) and positive (manganese- or sulfur-containing materials) electrode active materials, a performance hardly attainable with conventional non-aqueous liquid electrolytes today. Some solid electrolytes offer the possibility of thermodynamic stability (e.g., at the Li|LLZO interface). In contrast, some others offer the possibility of better kinetic stability by removing processes such as interface dissolution into a liquid or throttling solvent mass transport to the electrode interface^79–81^. However, in certain conditions, solid-state electrolytes can also become electrochemically active^74^. Thus, it is paramount to evaluate the electrode|solid electrolyte interaction during the development of all-solid-state batteries^82^.

##### Organic semi-solid and solid-state electrolytes

In the organic solid electrolyte category, we include commercially available, gel-type poly(vinylidene difluoride-co-hexafluoropropylene) (PVDF-HFP) electrolytes and gel-type poly(ethylene oxide) (PEO)-based electrolytes, such as those commercialized by Bolloré^83^. Although this company launched a pilot car-sharing program in North America, Europe and Asia to use this cell technology in electric city cars, this kind of lithium-metal-polymer (referred to as LMP®) battery never reached the mass market adoption in passenger cars^84^. One factor contributing to its poor commercial adoption is that they can only be used at relatively high temperatures (50 to 80 °C)^85^ and in a low voltage range (up to 4.0 V vs Li/Li^+^)^52^. However, these batteries are now deployed in commercial vehicles like the Mercedes eCitaro city bus^85^. To the best of our knowledge, there is no demonstration of prototype cells (e.g., at TRL 5) that work at room temperature (i.e., at around 25 °C) using a purely solid-state polymer electrolyte.

The semi-solid category includes highly viscous electrolytes, such as solvent-in-salt mixtures, i.e., electrolyte solutions with salt concentrations higher than the “standard” 1 M, which can reach as high as 4 M concentration or saturation points. A point of concern for concentrated electrolyte mixtures is the relatively high content of fluorinated salts, which also brings into question the lithium content (i.e., kg_Li_/kWh_cell_) and environmental impact of such a class of electrolytes. Indeed, a holistic approach to understanding opportunities for commercialization would also require a comprehensive life cycle analysis. It is also important to consider semi-solid electrolytes that can be prepared using commoditized chemicals. They could be easier to integrate into EVs versus cells comprising components that remain under development, such as ceramic separators.

##### Hybrid electrolytes

Concerns about the manufacturability and scalability of solid-state electrolytes and requirements on stack pressure continue to motivate the development of cell designs also incorporating non-aqueous liquid electrolyte solutions in hybrid solid-liquid configurations. Liquids can be employed to improve cell performance, e.g., by decreasing interfacial resistance or improving particle contact and Li-ion conductivity^86^. Hybrid solutions include solid-state cells using a mix of inorganic and organic electrolytes, as researched and proposed by several start-up companies that employ “catholytes” (i.e., electrolytes confined to the vicinity of the positive electrode) to enhance battery performance^87,88^.

##### General considerations for commercial development of electrolytes

One of the greatest opportunities that solid electrolytes present is to improve safety, energy, and extend cycle life, e.g., by increasing the voltage stability window in synergy with the electrode active materials. However, evaluating the introduction of alternative liquid- or solid-state electrolytes should be done carefully^23^.

Whenever a solid-state electrolyte layer is considered for cell production, its manufacturing is not a trivial process. Indeed, regardless of the battery chemistry, it is necessary to fabricate dense (~100%), non-porous, and thin (e.g., <20 µm) solid electrolyte films at a high yield (e.g., >30 m/min)^72^. Laboratory-scale type cells generally consist of solid-state electrolyte pellets (or membranes) hundreds of microns thick produced via non-scalable manufacturing techniques using single-side coated electrodes. These solid-state cells hardly represent the performance needed of a 10–100 Ah cell, which is considered the required target for EV-grade cells.

A solid-state electrolyte generally acts as a separator, and its weight and thickness (both larger compared to liquid electrolyte-filled polyolefin-based Li-ion cells separators) are crucial variables that must be tuned to reach specific energy and energy density of ≥350 Wh kg^−1^ and ≥900 Wh l^−1^, respectively, as expected for the first generation of commercial products. For both liquid- or solid-state electrolytes, it is crucial to test cells using realistic electrolyte loadings, doable from TRL 4, and to provide clear safety and performance testing of scaled-up prototypes, e.g., at TRL 5 or 6, both at the beginning and end-of-life, and different SOC.

Comprehensive safety testing is key to achieving higher TRL, as batteries always present a certain degree of safety-related risk. Solid-state electrolytes are not necessarily incombustible since some polymer and inorganic electrolytes can react with oxygen or water, generating heat and toxic gases, posing both a chemical and an explosion risk^74^. The amount of energy that can be released by a battery in single-cell format is a function of several factors, but primarily of the electrical and thermal energy stored. A holistic, system-level view and safety testing are ultimately required, as in the event of a fire, plastic, casing and pack materials could contribute to uncontrolled combustion.

It is also essential to provide a clear description of the thermal and mechanical requirements, e.g., applied stack pressure to make these cells work at room temperature and ideally in an extended temperature range (e.g., −30 to 100 °C) to compare with state-of-the-art lithium-ion batteries. Ultimately, it is necessary to understand the implications of integrating multiple single cells into a larger and more complex battery system (Fig. 3).

### Negative electrodes

While there have been steady advances in the performance of positive electrode materials used in lithium-ion batteries over the past 30 years, the negative electrode active material used in commercial cells has remained relatively unchanged^89,90^. However, various negative electrode active materials have been proposed for use in lithium-ion batteries; these materials are broadly summarised in Supplementary Fig. 2.

#### Insertion-based negative electrodes

Natural and artificial graphites are the most commonly used negative electrode active materials in commercial Li-ion batteries^91^. Natural graphite is obtained from mining and refining processes, while synthetic graphite is artificially prepared via high-temperature pyrometallurgical processes^91^. In recent years, an increasing amount of artificial graphite has been used alongside natural graphite in negative electrodes^91–94^. Natural graphite is a cost-effective material capable of delivering a specific capacity close to its theoretical value of 372 mAh g^−1^ at moderate specific currents (e.g., 100 mA g^−1^). In contrast, artificial graphite is more expensive and has a slightly lower specific capacity, but it enables a longer cell cycle life^95^.

Lithium titanate (LTO) has been used as an alternative to graphite in high-power applications. However, its adoption has been limited due to its high cost per energy unit and low energy density. LTO’s higher operating potential, around 1.5 V vs Li/Li^+^, with a voltage cut-off above 1.0 V vs Li/Li^+^, minimizes low-voltage degradation at the negative electrode|electrolyte interface. However, at the cell level, the low specific capacity (i.e., 170 mAh g^−1^)^96^ and a low nominal discharge voltage (limited to around 2.3 V) of LTO-based negative electrodes limits cell specific energy <100 Wh kg^−1^ and energy density <200 Wh L^−1^ when coupled with NMC-based positive electrodes and “standard” 1 M non-aqueous liquid electrolytes.

Beyond LTO, companies such as Toshiba^97^, Echion Technologies^98^ and Nyobolt^99^ are looking at innovating this cell concept with similar materials. These new cell chemistries could find a niche in applications such as hybrid vehicles, e.g., for heavy-duty applications. For example, niobium-based negative electrodes, although still at TRL 5^100^, can have capacities as high as 225 mAh g^−1^ at 34.3 mA g^−1^ and promise average cell discharge voltages of 2.3 V, which would result in higher energy densities than LTO-based cells^101^, but still lower than graphite-based cells. A near-monopoly of Nb supply could pose a risk to adoption^102^, and it is important to consider which technique is used for ore refinement and Nb purification^103^. Similarly to LTO, commercial adoption of these cells could be hampered by the higher $ kWh^−1^ cost compared to cells with graphite-based negative electrodes. However, as these technologies mature, end users of batteries could be willing to pay a higher upfront cost to access the performance requirements demanded by their specific application, in this case, power and cycle life, currently not achieved with graphite-based cells.

#### Conversion-alloy and alloy-based negative electrodes

Another important class of materials are alloys and conversion-alloys, first commercialized in a battery called “Nexelion” by Sony in 2005^9,104^, employing a negative electrode incorporating amorphous Sn-Co nanoparticles. Despite this high-TRL cell not being a commercial success, the development attracted research interest in alloy-based negative electrodes^89^, such as silicon-based materials^104^.

Commercially available lithium-ion cells are now beginning to use an increasing amount of silicon in the negative electrode in the form of silicon oxide, SiO_x_^91,105^, where the high theoretical specific capacity of silicon (up to 3579 mAh g^−1 94^ based on the mass of silicon) allows for improvements in energy density at the cell level even when silicon compounds only comprises a small fraction of the negative electrode (e.g., 2–10 wt. %^105,106^). However, this generally results in a trade-off with cycle life. Although there are no detailed accounts of who first commercialized silicon oxide in lithium-ion cells^2^, the material has been found in commercial cells manufactured as early as 2013, e.g., by Samsung^105,107^, and Tesla, which was the first major automaker to include silicon, as silicon oxide, in EV batteries^92^. Today, the percentage of silicon oxide in graphite-based negative electrode materials is generally estimated at around 2–10 wt. %^105,106^.

Industry is working towards a gradual increase in silicon content in the negative electrode, with GAC Motors claiming to be close to commercializing higher silicon content battery packs^108^. Companies such as Umicore^109^ have been developing the technology for over ten years. Umicore claims that the next steps include the “activation” of SiO_x_ using lithium or magnesium to increase initial cycle efficiency. Further steps include the introduction of silicon-carbon (Si-C) composite materials in the negative electrode, with blended graphite/Si-C electrode active materials having capacities in the range of 500–550mAhg^−1^_(active material)_^109,110^, values that suggest a moderate amount of silicon, around 10 wt. %^109^^,^ (we consider a moderate amount of silicon up to 20 wt. %). In parallel, several start-ups, collaborating with suppliers and automotive OEMs^29,111–114^, have been developing silicon-rich or silicon-dominant negative electrodes, i.e., up to 20–100 wt. %, in which the largest capacity contribution comes from silicon. Although some of these materials have been commercialized in niche applications, such as consumer electronics^115^ or aviation and aerospace^116^, no player has officially reached TRL 6 for supplying the automotive sector. Companies working on silicon-dominant batteries are generally expected to reach TRL 6-7 by 2025^29,111–114^.

Research on silicon-based negative electrodes focuses on buffering or reducing material volume changes upon lithiation and decreasing irreversible capacity loss during cell formation (e.g., via pre-lithiation) and cycling^109,117,118^. These drawbacks can be mitigated through several different approaches. Strategies include silicon-rich, monolithic or 3D-structured electrodes, such as those proposed by Enevate^119^, and negative electrodes prepared by vapour deposition, as developed by LeydenJar^120^. Vapour deposition can be used to grow silicon fibres and nanowires. Startup Amprius has used vapour deposition to deposit silicon on carbon nanotubes; this negative electrode material has been used in 3–10 Ah pouch cells^121^ with energies between 360–500 Wh kg^−1^, 890–1400 Wh L^−1^, and cycle life between 200–1,200 cycles, with fast charging capability^121^. Pure silicon nanowires can also be grown by vapour deposition; startup OneD Battery Science is taking this approach to grow silicon nanowires on graphite^122^. Various (nano-)structured, porous or templated silicon-based active materials, which could be integrated into standard lithium-ion manufacturing, are also considered and referred to as ‘drop-in’ technologies (e.g., by slot-die coating), such as those of Group14^114^. Automotive cells using silicon-rich anodes with up to 30 wt. % silicon are at TRL 5 today, with A-samples being sent to automakers. We estimate that automotive cells using >30 wt. % silicon are at TRL 4.

Unlike changing the positive electrode material, silicon-rich negative electrode active materials may require a significant redesign of the negative electrode and electrolyte system^60,123^, such as introducing new binders and new electrolyte additives. Hence, silicon-rich negative electrode materials can be considered a step change compared to the gradual improvements represented by using SiO_x_^123^.

#### Lithium metal-based negative electrodes

In the last five years, there has been a move towards the commercialization of rechargeable cells with lithium metal anodes, which have been proposed since the 1980s^9^. A variety of different concepts, such as (lithium metal negative electrode)|(sulfide electrolyte), (“anode-free” negative electrode)|(oxide electrolyte), (lithium metal negative electrode)|(polymer electrolyte), (lithium metal negative electrode)|(ionic liquid electrolyte), and many more, are also currently under development by several start-up companies, battery suppliers and automotive OEMs^9^.

**Fig. 4:**
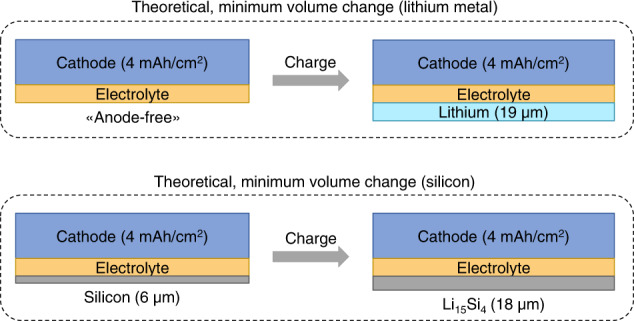
Theoretical volumetric changes upon cell charge of lithium metal (top) and silicon-based (bottom) cells. Volume change is visualized as a change in one dimension, namely thickness. In general, materials can expand in all three dimensions. The top panel shows that the deposition of 4 mAh/cm^2^ of lithium metal would lead to an increase in cell thickness of about 19 µm per negative electrode layer, based on a specific capacity of 3860 mAh/g and a density of 0.53 g/cm^3^, i.e., a volumetric capacity of 2045 mAh/cm^3^. The bottom panel shows that at the end of charge, the same amount of lithium (i.e., lithium equivalents) in an alloying reaction with silicon to form Li_15_Si_4_ would lead to an increase in cell thickness per negative electrode layer of 12 µm, and a comparable overall negative electrode thickness of 18 µm. A density of 2.33 g/cm^3^ was used for pure silicon and a volumetric capacity of 2194 Ah/cm^3^ for Li_15_Si_4_. Positive electrode and electrolyte layer are assumed to have a constant thickness. Volumetric capacity and density determine cell energy density, affecting how much space a cell would occupy, e.g., in a battery pack. Increasing cell energy density can allow, for example, more electrode layers or cells to be integrated into the same space.

Concepts using a negative electrode where no lithium metal is present during cell assembly and is extracted solely from the positive electrode on the first charge are often referred to as “anode-free”^124^. These present the most advantages from an energy perspective and the largest challenges for cell cycle life since any unwanted side reaction directly leads to a loss of capacity in the cell. “Anode-free” cells are also subject to larger volume fluctuation between charge and discharge (i.e., reversible and irreversible cell swelling, also termed “breathing”)^125^, which can require high stack pressures, and also lead to complex integration at the battery pack level. However, lithium metal’s low density (0.534 g cm^−3^ at 25 °C) means that silicon, with a density of about 2.33 g cm^−3^ at 25 °C, does not necessarily carry any penalty from an energy density perspective (Fig. 4).

For this purpose, it is worth considering the theoretical uniaxial volume change of lithium and silicon (Fig. 4). Both materials, upon lithiation, can undergo reversible cell stack volume changes of 10–20% (e.g., considering a positive electrode thickness of 100 µm and an electrolyte thickness of 20 µm or lower), which needs to be considered when battery cells are assembled and cycled in a battery pack. This requires a volume buffering strategy to be in place. Interestingly, if only the theoretical volume change is considered, lithium- and silicon-based cells can experience different magnitudes of swellings but can have comparable energy densities. With a minimally viable N/P ratio of 1, where the relative volume change would be highest^89,126–128^, a silicon electrode would be expected to exhibit a uniaxial volume change of 280% and an energy density of 2194 Ah cm^−3^ at the fully charged state^89,126^. The uniaxial volume change for lithium negative electrodes is higher than for pure silicon, as lithium metal has a lower density than that of lithiated silicon.

**Fig. 5:**
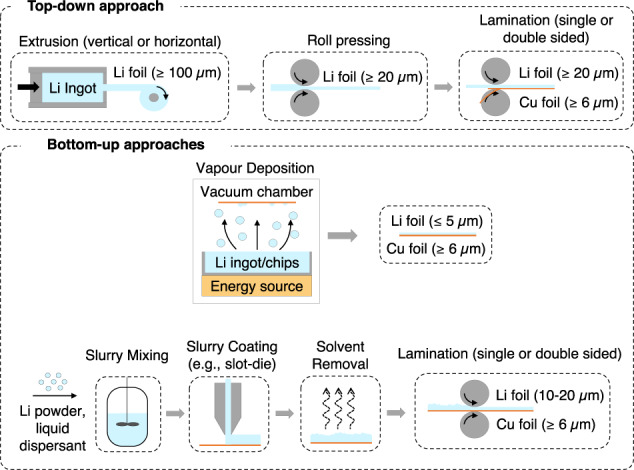
Production of lithium metal foils and electrodes. Top) Top-down method, i.e., extrusion of lithium metal ingots to produce lithium metal foils with a minimum thickness of 100 µm. Thickness can be reduced to a minimum of ca. 20 µm by roll pressing. The foil can then be laminated on current collectors, such as copper. Bottom) Bottom-up approaches. The upper part of the bottom panel depicts a simplified scheme of a physical vapour deposition method for producing lithium foil. A lithium source, such as an ingot or chips, is placed in a vacuum chamber. Mechanical, electromagnetic, or thermal energy is then applied to the lithium source to vaporize the metal, which is deposited on a current collector, such as copper, to act as an electrode. The lower part of the bottom panel depicts a method for lithium ink deposition, where stabilized lithium particles are dispersed in a liquid (slurry mixing), and the slurry is coated on a foil and dried. The lithium metal electrode can then be thinned and laminated to homogenize and flatten the surface.

Manufacturability is an open issue that needs to be solved to enable the use of lithium metal electrodes for the battery industry (Fig. 5)^129^. Conventional lithium metal foil manufacturing (Fig. 5 top, top-down approach), generally carried out under a dry or inert atmosphere (which can add to processing costs), includes an extrusion process, and leads to foils with a minimum thickness of 100 µm^130,131^. This thickness constitutes a large excess at the cell level (100 µm ≈ 21 mAh cm^−2^), particularly considering that the active lithium is generally already contained in the positive electrode material, with the cell assembled in a discharged state. A roll pressing procedure is commonly employed for thinning lithium metal foils. Currently, state-of-the-art processes produce foils with a minimum thickness of 20 µm and require the use of processing lubricants^131,132^.

Moreover, freestanding lithium foil can be complex to handle due to lithium’s mechanical properties, particularly ductility and adhesion^130,131^. Lithium metal can be laminated on current collectors such as copper or stainless steel foils to increase the negative electrode mechanical, electrical, and thermal properties. Current collectors are generally metallic foils that are mechanical support to deposit thin films on and act as electric current carriers^132^. With lithium metal being a soft, highly reactive material, all of these steps are non-trivial. To the best of our knowledge, there are currently no manufacturing plants capable of scaling-up lithium metal foil production for large-scale (e.g., EV-grade) cell manufacturing.

Bottom-up approaches (Fig. 5 bottom) include techniques such as physical vapour^133,134^ or ink depositions^135^. Vapour deposition borrows technologies either from the semiconductor or thin-film battery industries. For this bottom-up approach, achieving high-quality, homogeneous lithium layers with high throughput can be challenging. However, vapour deposition is well-versed to minimize lithium excess where thin layers (<10 µm) can be deposited^133–135^. Ink deposition is proposed by some suppliers, such as Livent^136^, but so far, the scalability and cyclability, particularly in large cell formats, still needs to be fully proven. Bottom-up approaches require a controlled atmosphere (e.g., low pressure and/or inert), and the resulting deposited lithium foil is expected to be highly reactive until the surface is passivated. These techniques can also be used for pre-lithiation (prior to cell assembly) of negative electrodes that do not contain lithium metal^118^.

Regardless of the production approach, the handling and particularly shipping of lithium metal represents an additional major barrier to the widespread adoption of the material^129^. Transport requires additional measures in accordance with regulations regarding the transport of dangerous goods, such as the Agreement concerning the International Carriage of Dangerous Goods by Road (ADR)^137^ and the International Air Transport Association (IATA) Dangerous Goods Regulations (DGR)^138^. Currently, shipping lithium metal requires large containers kept under a controlled inert atmosphere^129^. Higher logistical costs or co-location of lithium foil manufacturing plants (e.g., adjacent to cell manufacturing plants) should therefore be considered when envisioning manufacturing lithium metal battery cells.

#### General considerations for negative electrodes

To summarize, there is no single solution to every technical concern related to lithium-based battery negative electrodes. Indeed, different cells present challenges that cannot be fully resolved at once; instead, a compromise between safety, energy content, cost and cycle life needs to be reached. So far, negative electrode improvements in large-scale batteries have been marginal: graphite is still the material of choice, although the inclusion of silicon as a composite with graphite is already happening at the commercial cell level.

Arguably the push for higher-energy batteries has led to rapid incremental developments of positive electrode active materials^139^, while research on negative electrodes tends to lag behind. This is partly due to companies that have developed positive electrode active materials successfully managing the industrial risk of bringing a new product to market^140^. Indeed, replacing graphite-based negative electrode material requires a “step-change”, meaning that the application for specific negative electrode chemistry needs to be considered by rethinking the whole system, i.e., with a holistic view of the cell, system integration, and practical manufacturability. This also implies an opportunity for a technology leapfrog. Companies developing these solutions are generally start-ups, many of which have now attracted large investments from automotive OEMs. This is possibly because start-ups are better placed to pursue high-risk projects and manage fast-paced development cycles, compared to large manufacturing and engineering firms.

### Positive electrodes

#### Insertion-based positive electrodes

LiCoO_2_, with a practical electrode-level specific capacity of ca. 135 mAh g^−1^ ^141^, was the first commercial positive electrode active material used in lithium-ion batteries^12^ and the first lithium-ion based electric vehicles (Nissan Prairie Joy EV, 1997)^142^. Despite the introduction of lower-cost materials into consumer electronics, like LiFePO_4_ and lithium manganese oxide (LMO), in 2008, Tesla used LiCoO_2_-based (LCO) positive electrodes in the cells used in its first EV, the Roadster^143^. These cells were available in a 18650 format and offered higher energy densities than other cells on the market at the time that used LFP or LMO as positive electrode active materials. 18650 LCO cells were also easier to procure due to their widespread use in laptop battery packs. However, as the electric vehicle market began to take shape, the automakers outside of China (which has the largest lithium-based battery manufacturing industry globally today)^11^ started to investigate the use of alternative cobalt-poor battery chemistries that better-suited EV requirements. At the time, this meant looking for positive electrode active materials that enable a higher energy content, with a lower raw materials cost, reasonable cycle life, and safety comparable to the standard LiCoO_2_-based electrodes.

This led to the emergence of nickel and manganese-based chemistries, such as NMC and NCA. These positive electrode active materials replace (partially or completely) expensive cobalt for cheaper nickel (prices true as of August 27, 2021)^144^. The raw materials used to produce Panasonic/Tesla’s Ni-rich (>90% nickel on a molar basis as a fraction of the transition metal in the positive electrode) NCA92 positive electrode chemistry are more than 50% cheaper than those in LCO on a kg basis^145^. By substituting cobalt with nickel, it is possible to increase the practical capacity of these positive electrode materials, as the equivalents of lithium extracted from the positive electrode active material increase from 0.6 up to 0.75–0.80^61^. However, this can also lead to accelerated structural deterioration^146^.

**Fig. 6:**
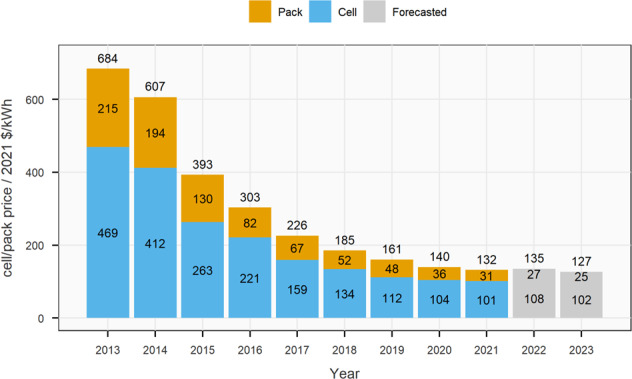
Battery Cell/Pack price forecast. The figure shows the real average decline in the battery pack and cell prices for lithium-ion batteries from 2013–2021. Prices are split between the cell and pack components. The 2022 and 2023 prices are forecasted prices based on expected changes to critical battery raw materials. The forecasted projections are based on the state of the market in November 2021^197^.

The current increase in raw material prices^147^ (true as of November 2022) creates problems for cell manufacturers and automotive OEMs at a time when they are trying to decrease the price of batteries and electric vehicles. Based on current forecasts, 2022 may be the first year since the widescale adoption of EVs started over a decade ago, that average lithium-ion battery prices increase (Fig. 6). This may influence OEM decisions when it comes to introducing new chemistries. For example, in 2018, when cobalt prices reached almost 100,000 $ t^−1^, companies quickly switched from high-Co-content to high-Ni-content (with the minimum possible Co content) NMC positive electrode active materials^148^. This was particularly evident within the Chinese battery industry, where NMC811 was introduced around two years earlier than anticipated before cobalt prices saw their rapid rise^149^.

Despite the higher energy of cells using high-Ni-content positive electrodes, for much of the last decade, Chinese companies favoured LFP. The drivers behind China’s initial focus on LFP are complex and outside the scope of this article, but it is heavily related to the legal battle for LFP licensing that concluded at the beginning of the last decade. After a couple of years of testing batteries with Ni-rich positive electrodes, encouraged by generous government subsidies that favoured the development of high energy density batteries and long-range EVs, Chinese cell manufacturers and automakers are again favouring LFP^150^. Chinese cell manufacturer BYD recently switched all its passenger EVs over to LFP using its Blade Battery technology^151^.

Concerns over battery costs and raw material supply have been drivers in this switch back to LFP. It has also been enabled by innovative cell and pack designs that improve the specific energy of LFP systems at the pack level while still benefitting from LFP’s low cost.

In the longer term, automakers and manufacturers still expect to deploy new positive electrode chemistries tailored to specific applications.

Some automakers are focusing their attention on high-Mn-content chemistries^152^, such as LNMO, manganese-rich NMC, and LMFP, e.g., for the volume vehicle segments^152^ as they balance raw material costs with vehicle range/performance, or for hybrid vehicles which will benefit from the high voltage, high power capability. To date, however, there is no clear evidence of battery cells with TRL > 5 containing these materials. For high-performance vehicle segments, automakers are still targeting Ni-based chemistries with an increasing nickel content and lower cobalt content. Finally, there is a concrete opportunity for cells based on NMC active materials with an intermediate Co content to be cost- and performance-competitive with those based on Ni-rich NMCs^153^ by increasing the upper voltage cut-off. This cell development trend has been observed for LCO-based consumer electronic batteries^141^. EV adoption, however, could be further in the future, as additional electrolyte and active materials’ developments and demonstration at scale are still required.

#### Conversion-based positive electrodes

In parallel, a range of positive electrode active materials are at an early stage of development (TRL 4). For example, Solid Power, a US start-up developing solid-state batteries, claims to have developed prototype cells using conversion-type positive electrode active materials such as FeF_3_ or FeS_2_^154^. These materials are being developed due to a theoretical capacity in the range of 700–900 mAh g^−1^, with a lithiation potential in the range of 1–2.5 V vs. Li/Li^+^^155,156^. If this class of materials (including also elemental sulfur or oxygen, or other non-lithiated positive electrode materials^157^) are eventually commercialized, they could result in a reduction in the mass of positive active material required per kWh of cells from 1–2 kg kWh^−1^ (with the current generation of insertion layered oxide) to less than 1 kg kWh^−1^ ^158^. While these materials could be considered attractive on this basis alone, it is worth mentioning that conversion-type materials have drawbacks, which could greatly hinder their practical exploitation. Drawbacks include: (i) capacity loss and large voltage hysteresis during cell cycling, (ii) poor power densities due to sluggish kinetics and multi-electron reactions, (iii) relatively high strain upon lithiation and delithiation, and (iv) need for a large amount of lithium metal in the negative electrode (i.e., potentially double the amount or more compared to cells using Li-based layered oxides positive electrodes). The lower average voltage of the positive electrode will require a higher capacity loading (in terms of mAh cm^−2^) that will lead to higher local current densities at the negative electrode and higher costs, particularly considering complexities with handling and shipping lithium metal foils. Moreover, cells would be assembled charged rather than discharged^157^. It is unclear if this could add to the complexity of cell manufacturing at a large scale.

### Challenges in scaling up Li-ion batteries

**Fig. 7:**
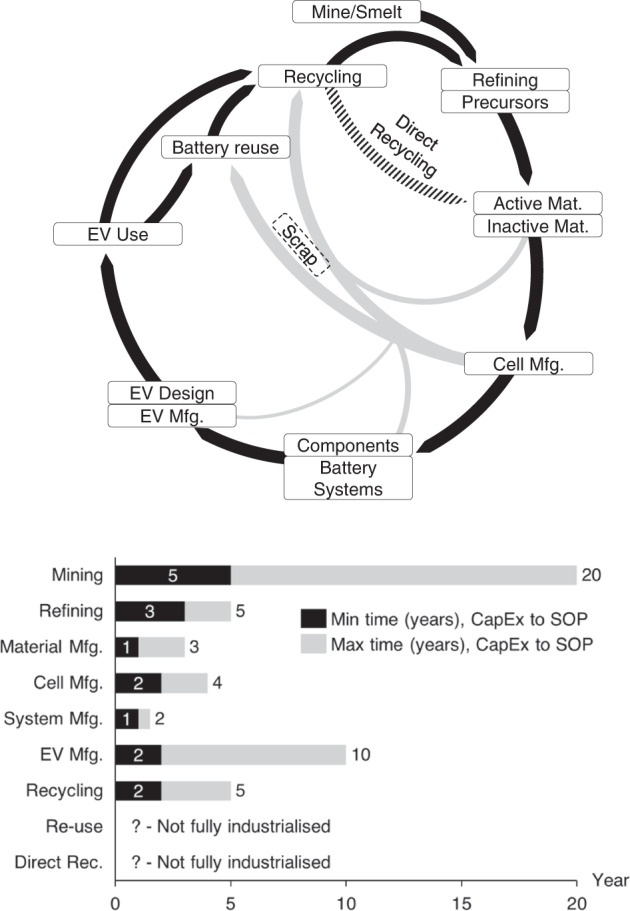
Circular EV battery value chain from mineral extraction to recycling (top), and typical time required to reach SOP (bottom). The time in years from CapEx to SOP is estimated from capital expenditure to start a project/plant to when production starts. We estimate both the typical minimum time (black bar), and maximum time (light grey). We assume technical maturity and further delays can be expected if the technology is not developed or there is a lack of know-how. Most steps require high-precision manufacturing and can have different degrees of complexity for market entry. The values are indicative, sourced from public announcements, and in-line with those disclosed by public organizations such as EIT InnoEnnergy^199^. For scrap, we assume that the largest volume will initially come from giga-factory ramp-up.

Lab-scale material development and engineering improvements can be the primary hurdles in bringing new technology to market. While challenges such as scaling material production from grams to tons are well understood, additional problems are often overlooked, such as the complex value chains, with dozens of suppliers required to source all the materials and components (see Fig. 7, top). Building a manufacturing plant can take several years to commission from capital expenditure (CapEx) to SOP (Fig. 7, bottom), and the time it requires depends on the product being produced. A chemical plant producing layered oxide positive electrode active materials will be very different from a plant that produces battery cells, which requires precision manufacturing and high automation to be cost-competitive. Here we use a series of examples to illustrate how supply chain considerations and poor cost assumptions can de-rail technology development.

#### The supply chain

Moving to positive electrode chemistries with high manganese content potentially offers a route to balancing manufactured cell costs with performance metrics such as specific energy^159^. A variety of established manufacturers and start-ups are pursuing these materials, e.g., Haldor Topsøe^160^ and Nano One Materials^161^ in the case of LNMO, BASF in the case of NMC 370, SVOLT^162^ in the case of NMx, and HCM^163^, SAFT^164^ and CATL^165^ in the case of LMFP or LxFP (with x an undisclosed number of different substituents, such as CATLʼs “M3P”^166^). These companies are advancing the large-scale production of, and claim to achieve, high-performing positive electrode materials^160,161^. However, the current battery-grade manganese supply chain is insufficient to support these technologies’ widespread adoption today. Indeed, current projections for manganese sulfate supply show that demand will outstrip supply as early as 2025 if chemical companies do not invest in additional capacity (see Supplementary Fig. 3). To prevent manganese sulfate availability from being a bottleneck, companies that plan to use these positive electrode materials will need to work closely with chemical suppliers to ensure that production capacity is ramped up in line with their requirements. These issues are not only a problem for the producers of the material but also potentially disruptive for the plans of end-users, such as Norwegian battery manufacturer Morrow^167^ (who have partnered with Haldor Topsøe to produce LNMO cells) and companies like Volkswagen who have indicated manganese-rich chemistries as a key part of their future plans^152,168^.

Batteries using inorganic solid-state electrolytes face similar supply chain constraints. There is no existing supply chain for cells using sulfide electrolytes (e.g., Li_3_PS_4_) to provide the required lithium sulfide materials. This means that companies have to develop their supply chains while also commercializing the batteries themselves. The supply chains of oxide-based solid-state electrolytes (e.g., Li_7_La_3_Zr_2_O_12_, LLZO) face similar difficulties. Lanthanum, as used in LLZO, was estimated to have an annual production of around 50,000 tons in 2019^169^. We estimate that 1 GWh of batteries using a 20 µm thick LLZO electrolyte with an 80 µm thick NMC811 positive electrode will require around 255 tons of lanthanum. Current lanthanum production could therefore support around 200 GWh of all-solid-state battery production.

The growing use of inorganic solid-state electrolytes and the application of pre-lithiation technologies and lithium metal negative electrodes promise to increase lithium demand significantly. If the rate of demand increase is not properly understood with cooperation amongst companies from across the value chain, this could lead to further material bottlenecks. It is already difficult to forecast future demand for lithium, and other battery raw materials, as forecasts for passenger EV sales and their associated lithium-ion battery demand vary wildly. In its 2021 electric vehicle outlook, BloombergNEF forecasted around 32 million passenger battery EV and plug-in hybrid EV sales annually by 2030^170^. In contrast, the International Energy Agency (IEA)^11^, for the same year, draws a few scenarios for EV sales. Their most conservative forecast is at >30 million EV sales by 2030 but expects that over 65 million EV sales would be needed in 2030 to meet the requirements of the 2050 Net Zero Emissions scenario^11^. This uncertainty alone creates difficulty in scaling up. However, material suppliers can de-risk this to some extent by working closely with their customers.

#### Cost forecasting

When developing new technologies, academic researchers or start-ups need to forecast the cost of the new system compared to the incumbent technology to justify commercialization, win funding and pursue development. This aspect requires multiple assumptions about existing manufacturing processes and supply chains and how they will change in the future. For academic researchers and start-ups, it can be difficult to get an accurate representation of what these costs are and how they will change. However, there are publicly available tools, such as BatPac^6^, which can be helpful. If the assumptions used are not reflective of the industry, then the cost forecasts could result in unrealistic expectations of the competitiveness of the final product. This, in turn, will damage the business case of start-ups or lead to funding being allocated to academic lines of research that are unlikely to result in technology improvements that will benefit the industry or result in technological advancements.

Difficulties in accurately forecasting production timescales can also damage the scale-up opportunities of new technologies. Overly aggressive timelines for introducing new technologies can make an investment attractive to naive investors, but in the end, may lead to a final product that is more expensive than the incumbent technology. For example, a new cell design may be commercialized on the basis that when produced, it will be cheaper than the incumbent. However, a delay in production could mean that gradual improvements to the incumbent cell design leads to the manufactured cost of the incumbent design passing below the forecasted manufactured cost of the new design. While seasoned investors may be more cautious than companies looking to raise capital, technology developers should be realistic about what is achievable. Overpromising and underachieving will cause more harm to the industry as a whole.

#### Manufacturing processes and system design

We have mainly discussed the advantages and drawbacks of introducing new materials into the battery industry. However, it can be equally difficult to introduce new manufacturing processes and techniques as well as electrode and cell designs^23^. In the manufacturing space, companies are exploring new processes such as pre-lithiation, dry electrode coating, and improved quality control processes. However, it is challenging to persuade cell manufacturers to adopt these technologies, which, when initially introduced, are likely to lower yields and increase CapEx. This generally leads to higher manufactured cell costs. Despite these challenges, some companies are trying to commercialize these technologies

Prominent examples include 24M’s “SemiSolid” cell design, which Norwegian cell manufacturer Freyr is adopting^171^ among others^172^. While 24M’s technology is being commercially adopted, it is notable that a major cell manufacturer has not licensed the technology but is instead being commercialized by a battery cell manufacturing start-up company, presenting venture on venture risk and reducing the likelihood of commercial deployments to some extent. In some respects, this should be expected for large-step changes in manufacturing, as established companies are typically more risk-averse than small start-ups. The promise of leapfrogging incumbents and gaining market share is often reason enough for a start-up to take on this technology risk.

Start-up companies such as EnPower and Addionics are also in the process of scaling and commercializing their proprietary electrode designs. These companies claim their products would enable the development of simultaneous high-power and energy devices. However, Addionics is yet to start large-scale pilot production (>100 MWh)^173^, and EnPower is having to scale pilot production internally to provide the volume of batteries required for customer qualification, requiring significant CapEx investment from the company^174^.

Finally, series, or bipolar, stacking^175^ is being actively researched and scaled-up by companies such as ProLogium^176^ and Toyota^177^. Advantages can include better thermal and electrical properties, and reduced packaging but at the expense of a more complex manufacturing process and system design.

The biggest system design adopted commercially over recent years is the so-called “cell-to-pack” design, such as BYD’s Blade Battery. These systems have been quickly adopted as they improve performance but do not fundamentally alter the chemistry of cells or require radically new manufacturing processes.

#### Qualification of parts in the automotive industry

Even with a mature value chain, supplying parts to the automotive industry is non-trivial, and the process can be time-consuming. Suppliers who wish to engage with the automotive industry must undergo a standardized, rigid qualification process, which is regulated at the international level (see, e.g., International Automotive Task Force, IATF 16949^178^). The most common automotive standards for part qualification are the German Verband der Automobilindustrie (VDA) production process and product approval (PPA)^179^ and the Automotive Industry Action Group (AIAG) Production Part Approval Process (PPAP)^180^.

Some considerations for serving the auto industry are discussed in the literature^181^, with guidelines available from governmental and automotive standard bodies^182^. For example, let us consider the supply of Li-ion battery cells to an automotive OEM for integration into a battery pack. In this case, battery cell suppliers, such as Samsung SDI, CATL, and LG Energy Solution, are expected to reliably supply safe, high-quality parts with minimum rejects, i.e., in a batch of cells supplied to an automotive customer, where less than 10 cells in a million (10 ppm) could be defective. Parts need to be rigorously tested using robust processes.

Following VDA guidelines^182^, qualification for new cells would start at the A-sample, a prototype cell at TRL 5. The A-sample cell does not need to be series produced, but it must be safe, functional, and close to the final design both in terms of performance and geometry: cell footprint and size are fixed. This prototype can compromise on lifetime and performance but should satisfy most of the requirements to lead to the qualification of B-samples, where the cell design is unalterable. Past the B-sample stage, the focus is on manufacturing. A larger number of trial modules/packs are assembled, and cells are series produced, which constitutes the C-sample stage (TRL 6). Finally, in the D-sample stage, the battery cells are produced at scale, ready to be implemented commercially, and ready to pass automotive part approval, e.g., undergo Production Part Approval (PPA) and reach TRL 7.

Testing requirements can increase ten-fold, from hundreds of cells for A-samples to tens of thousands for C-samples. The type of tests required includes performance and safety, with the latter being a strict requirement at any stage. Tests are also rigorously defined in standards, guidelines and regulations (such as by the International Electrotechnical Commission, IEC 62660, by the United Nations, UN38.3, UN ECE R100^181,183^) or routine testing (e.g., United States Advanced Battery Consortium LLC, USABC, guidelines)^184^. It is essential to understand that most actors, academic or industrial, particularly during the initial stage (where start-up companies are usually involved), lack the resources to accurately carry out these tests or enter the supplier qualification step for the automotive segment. A lack of appropriate process control can also result in manufacturing defects, potentially leading to costly product recalls^2,185^.

## Summary and recommendations

Taking into account all the various aspects of battery research discussed in this perspective article, we summarize below the main take-home messages that we hope could be useful for expert, non-expert, academic and industrial researchers when evaluating claims in the field of lithium-based secondary batteries and, energy storage research in general.

Remarkable improvements to cost and performance in lithium-based batteries owe just as much to innovation at the cell, system and supply chain level as to materials development. Battery development is an interdisciplinary technical area with a complex value chain. For academic research to provide the largest benefit to these sectors, there needs to be collaboration across disciplines, with the industry actively advising academia on specific end-customer requirements. This could be fostered, for example, by supporting industrial researchers taking shared positions with academia, encouraging industrial researchers to publish more peer-reviewed papers, and increasing academic representation at industry conferences (and vice versa).

Metrics are important, but which metrics matter and how they translate from theory to system is case-dependent. A clear consideration of the bigger picture is vital for effective applied research. We have evidenced how the high theoretical energy density/specific energy of a positive electrode active material, like NCA, does not necessarily translate to higher performance at the pack level. Many KPIs need to be considered when scaling a material, as a battery with high energy and low cycle life could have limited applications. All KPIs need to be evaluated for devices at high TRL, and manufacturing itself can be the biggest challenge, particularly when innovative technologies are not “drop-in”. In cell developer QuantumScape’s recent earnings call, when asked if the company needed to make perfectly uniform and totally defect-free solid-electrolyte-based separators for its cells to work, CEO Jagdeep Singh hinted at these challenges when he replied, “the key is knowing which defects matter and which ones don’t and to focus on the former”^186^.

Moving up in the TRL scale is an increasingly expensive and complex task. The ability to reach TRL 9 requires an understanding of many requirements and a quick transition across lower TRLs. It is easy to over-simplify the factors involved in commercialising a technology, subject to a vast and continuously changing global industry that naturally introduces uncertainty into economic viability. It is perhaps too easy for academic researchers to be overly optimistic about the ability of a certain technology to scale based on, for example, preliminary performance data or raw materials costs, unaware of the exponentially increasing requirements on resources required to bring a new technology to market. This is perhaps best exemplified by Tesla’s chief executive officer Elon Musk’s comments regarding “the machine that builds the machine”, which references the difficulties companies face in manufacturing at scale^187^.

Hype, over-extrapolation and perverse incentives only risk harm to the sector in the long run, and all participants should take responsibility for fostering good communication and best practices. Within academia and industry alike, the battery field has unfortunately cultivated a reputation for hype, false promises and unrealistic goals. Many other scientific areas have had to grapple with reproducibility or scientific integrity crises in recent years, brought on by shortcomings which can just as easily be found in the battery scientific literature. In this regard, the whole battery research community must support initiatives such as the adoption of standardised testing protocols, standardisation of data collection, and requirement of publishing raw data. Such developments promote transparency and transferability of knowledge, especially considering the increasing importance of research approaches based on machine learning or, more broadly, artificial intelligence.

In particular, we strongly recommend that battery researchers keep in mind the following aspects to improve material development without neglecting the practical application aspect:

1. The electrolyte effectively sets the electrochemical energy storage system boundaries, including safety and cycle life, and electrolyte development is an exercise in compromise. For example, cost has to be balanced with electrochemical stability and ionic conductivity. Improvements in cycle life are key for most applications, and research on new electrolyte systems should be incentivised.
2. In recent years, the focus of the industry, and particularly automakers, has been on achieving a step change in energy density, which has sharpened the focus on introducing or switching to silicon and lithium metal negative electrodes, thus, necessitating a re-thinking of cell design. These new concepts must, of course, meet minimum performance requirements. However, the continued improvement to what could be considered ‘legacy’ battery concepts, as well as increasing raw material costs, have seen some companies achieving competitive performance from such ‘legacy’ systems as graphite | |LFP batteries. Further improvements in these battery systems could open up the possibility of business model innovations, such as vehicle-to-grid (V2G) integration.
3. Targets in terms of cost reduction and increased energy and lifetime can also be achieved with incremental improvements, e.g., by refining pack design and manufacturing processes such as BYD’s Blade battery and pack, but also active and inactive materials, e.g., electrolyte and additive optimisation as highlighted by Professor Jeff Dahn (Dalhousie University)^188^.
4. Positive electrode active materials generally differentiate lithium-based batteries, and choice is driven as much by cost as by performance; this is likely to continue in the short to medium term. In the future, negative electrode material choice could similarly differentiate these batteries.

We would also remark on the strategic role of the supply chain. This area is crucial in reducing cost and improving lithium-based batteries’ performance while strongly influencing the manufacturing and material production processes. Another equally important area is the need for data-driven environmental sustainability analysis, such as life cycle assessments, to understand the environmental impact of batteries from raw-material mining to recycling.

As an increasing number of researchers with various scientific and technical backgrounds turn their focus to the battery industry, it is important that they acquire a broader view of the research and development landscape across the sector, not narrowing their vision to only focus on their field of expertise. In doing so, it is possible to avoid reaching misleading or ineffective conclusions that fail to advance the scientific understanding and progress of lithium-based batteries.

In this regard, we consider the growth of the online battery community during COVID−19 as an encouraging development. Hybrid conferences can be effective forums for experts and non-experts to engage with each other and acquire a broader view. Open, inclusive, and cost-effective initiatives should be incentivized, starting from free access to scientific research and including accessible communication platforms with academic and non-academic participation, such as the Battery Modelling Webinar Series^189^, Battery Brunch^190^, and Battery Pub^191^. However, these initiatives come with some challenges and limitations, such as (i) a risk that misinformation may spread (moderators are needed); (ii) open data can be misused by entities with a conflict of interest or misinterpreted by non-experts; (iii) risk of communities becoming self-referential;^192^ (iv) confidentiality issues, where researchers working closely with industry can be restricted by non-disclosure agreements. In addition, many scientists have found social media platforms, such as Twitter or LinkedIn, valuable venues for networking and outreach^193^.

A more rigorous approach to science is ultimately needed. The end goal should be accelerating innovations that directly improve battery systems and increasing the number of relevant, reproducible, and openly accessible peer-reviewed scientific articles. This is particularly important considering that the amount of time and non-time resources needed to drive the energy transition are finite^194^.

Nowadays, there is too much research that confuses, rather than adds to, progress, and we need joint action from stakeholders, industry, academia, and publishers to solve this issue. Resources should not be squandered on the basis of (often unknowingly, potentially in good faith) biased and/or unreliable studies or well-sounding press releases. Indeed, a more critical, engineering-led, numerical, and transparent approach to scientific research is certainly required.

As a closing message, the reader should bear in mind that transparency is a key requirement, and the lack of adequate, impartial, and exhaustive communication is usually the main reason for the divide between academia and industry or, more broadly, for the failure of collaborative research activities.

## Supplementary information

Supplementary Information

## Data Availability

Data is fully available on request from the authors
