# Supplementary material for: A non-academic perspective on the future of lithium-based batteries
Source: Nat Commun. 2023 Jan 26;14:420. doi: 10.1038/s41467-023-35933-2 (PMC9879955; doi:10.1038/s41467-023-35933-2)
Supplement: Supplementary file 1 — Supplementary Information [file 41467_2023_35933_MOESM1_ESM.pdf]

Supplementary Information

A non-academic perspective on the future of lithium-based batteries

James T. Frith[1, *, †,**] Matthew J. Lacey[2, *, †] Ulderico Ulissi[3, *, †, ***]

1 Volta Energy Technologies, 28365 Davis Pkwy, Warrenville, IL 60555, United States

2 Scania CV AB, 151 87 Södertälje, Sweden

3 Sphere Energy SAS, 250 Bis Boulevard Saint Germain, 75007, Paris, France

* corresponding authors: james.frith@volta.vc, matthew.lacey@scania.com, ulderico.ulissi@live.com

† these authors contributed equally to the work.

** the author is based in London, UK

*** the author is based in Birmingham, UK

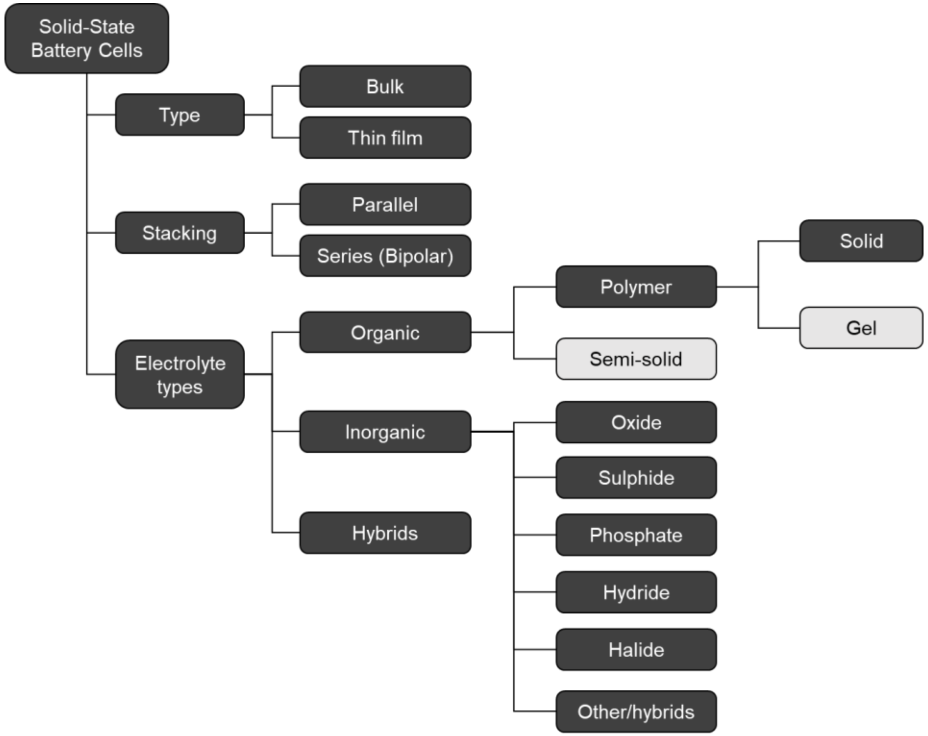

Supplementary Figure 1: Solid state battery categories. The categories are based on internal construction (type), stacking configuration and electrolyte chemistry. Semi-solid and gel electrolyte are highlighted (white boxes), as these should be considered as viscous fluids rather than purely solid-state. Stacking refers to the internal battery structure, where parallel stacking is the structure of a conventional, state-of-the-art lithium-ion battery.

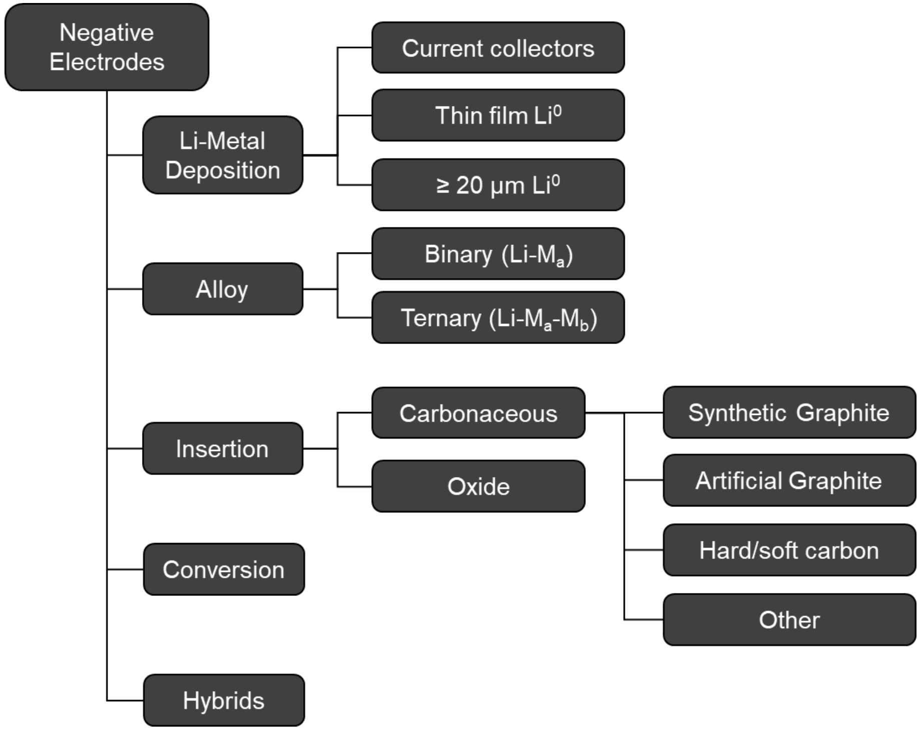

Supplementary Figure 2: Negative electrode active material categories. The categories are based on the main lithium reaction mechanisms. In hybrid, we include materials in which the reaction is not dominated by a single mechanism, e.g., conversion-alloy.

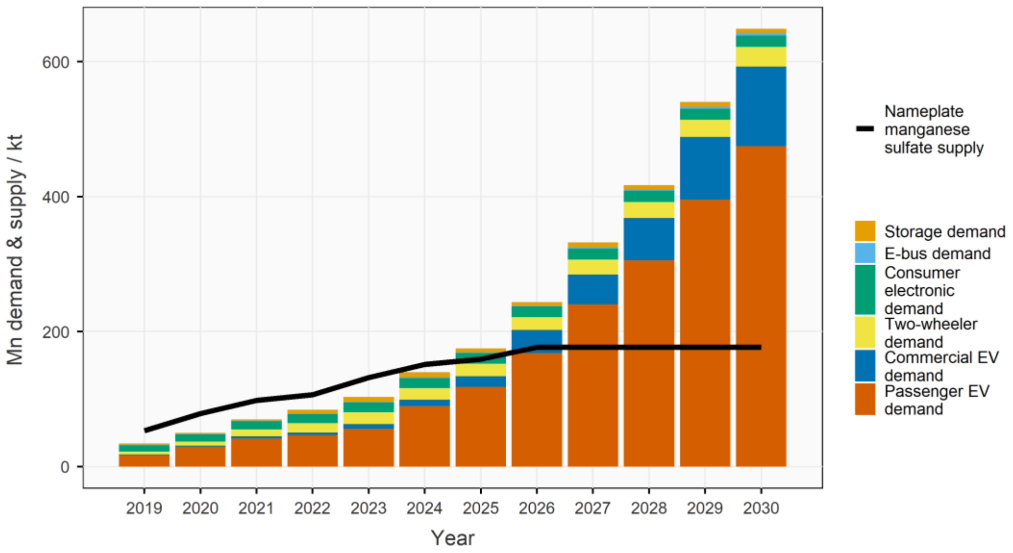

Supplementary Figure 3: Supply and demand balance for manganese sulfate. Demand, in kt of manganese sulfate from 2019 (actual) to 2030 (forecasted) is reported per year, as coloured bars, with each colour identifying a specific application, i.e., stationary storage, transportation (E-bus, two-wheeler, commercial EV, passenger EV) and consumer electronics. Supply in kt of manganese sulfate from 2019 (actual) to 2030 (forecasted) is reported as a black line. Data source: BloombergNEF, Global Manganese Outlook 2020-2030.

Supplementary Table 1: List of commercially deployed Li-ion battery cells and relevant specifications used to create the energy plot presented in the main text as left panel of Figure 2. Specific energy values are calculated dividing the energy content (Wh) by the mass of the cell (g). Energy density values are calculated dividing the energy (Wh) divided by volume. The latter is calculated as depth*width*height for prismatic and pouch cells, and π*(depth/2)^2*height for cylindrical cells.

Name	Cathode	Anode	Format	Capacity / Ah	Energy / Wh	Mass of cell / g	Width / mm	Height / mm	Depth / mm	Source
CATL 60 Ah	LFP	Gr	Prismatic	60	192	1821	217	135	29	https://www.indiamart.com/proddetail/catl-type-60ah-3-2v-15000-cycles-22154610991.html
CATL 161 Ah	LFP	Gr	Prismatic	161	515.2	3100	280	82	62	https://batteryfinds.com/wp-content/uploads/2021/06/CATL-161Ah-Lithium-Iron-PhosphateLiFePO4-LFP-Battery-Cell-Product-Specification.pdf
CATL 302 Ah	LFP	Gr	Prismatic	302	972.4	5556	207	174	72	https://deligreen.en.made-in-china.com/product/QOzGyZpdfrVc/China-Grade-a-Catl-3-2V-302ah-LiFePO4-Cell-300ah-310ah-320ah-Lithium-Iron-Phosphate-Battery-for-Solar-RV-Inverter.html
ACE 50 Ah	LFP	Gr	Prismatic	50	160	860	148	129	26	https://www.acebattery.com/products/prismatic-cell-lifepo4.html
ACE 100 Ah	LFP	Gr	Prismatic	100	320	1800	173	133	47	https://www.acebattery.com/products/prismatic-cell-lifepo4.html
ACE 200 Ah	LFP	Gr	Prismatic	200	640	4100	207	173	53	https://www.acebattery.com/products/prismatic-cell-lifepo4.html
ACE 277 Ah	LFP	Gr	Prismatic	277	886.4	5200	207	173	71	https://www.acebattery.com/products/prismatic-cell-lifepo4.html
Gotion ESS105	LFP	Gr	Prismatic	105	336	2030	200	175	27	https://www.shanghai-electric.com/group_en/c/2019-08-12/557924.shtml
Gotion ESS90	LFP	Gr	Prismatic	90	288	1980	200	175	27	https://www.shanghai-electric.com/group_en/c/2019-08-12/557924.shtml
Gotion ESS96	LFP	Gr	Prismatic	96	307.2	2020	200	175	27	https://www.shanghai-electric.com/group_en/c/2019-08-12/557924.shtml
Gotion ESS100	LFP	Gr	Prismatic	100	320	2050	200	175	27	https://www.shanghai-electric.com/group_en/c/2019-08-12/557924.shtml
Gotion ESS40	LFP	Gr	Prismatic	40	128	925	148	115	28	https://www.shanghai-electric.com/group_en/c/2019-08-12/557924.shtml
Samsung CS1200R	NMC	Gr	Prismatic	123.9	456.8	2228	174	125.5	45	https://www.batemo.de/products/batemo-cell-library/cs1200r/
Lishen 51 Ah	NMC	Gr	Prismatic	53.1	195.8	911.4	147.7	97.5	28	https://www.batemo.de/products/batemo-cell-library/c-pr51a/
LG A7	NMC	Gr	Pouch	51.1	188.7	895	298	153	10.6	https://www.batemo.de/products/batemo-cell-library/a7/
LG E63B	NMC	Gr	Pouch	61.1	225.2	887.8	310	114	15	https://www.batemo.de/products/batemo-cell-library/e63b/
LG E66A	NMC	Gr	Pouch	63.5	232.2	897	350	104	11.7	https://www.batemo.de/products/batemo-cell-library/e66a/
Samsung 94 Ah	NMC	Gr	Pouch	94	345.92	2010	173	125	45	https://pushevs.com/2018/04/05/samsung-sdi-94-ah-battery-cell-full-specifications/

Panasonic Tesla Model 3	NCA	Gr-Si	Cylindrical	4.66	17.1	68.5	21	70		https://www.batemo.de/products/batemo-cell-library/tesla-model-3/
LG INR21700 M50	NMC	Gr-Si	Cylindrical	5.013774105	18.2	68	21	70		https://www.dnkpower.com/wp-content/uploads/2019/02/LG-INR21700-M50-Datasheet.pdf
A123 ANR26650M1B	LFP	Gr	Cylindrical	2.5	8.25	70.6	26	65		https://a123batteries.com/anr26650m1-b-lithiumwerks-nanophosphate-3-3v-2-5ah-lithium-iron-phosphate-battery/
A123 APR18650M1B	LFP	Gr	Cylindrical	1.1	3.63	39	18	65		https://www.batteryspace.com/prod-specs/6612-APR18650M1B.pdf
Oxis High Power	Sulfur	Li	Pouch	19	40.85	141	151	116	10.7	https://45uevg34gwlltnbsf2plyua1-wpengine.netdna-ssl.com/wp-content/uploads/2019/07/OXIS-Li-S-Ultra-Light-Cell-spec-sheet-v4.2.pdf
Oxis High Energy	Sulfur	Li	Pouch	14.7	31.605	85	145	78	10	https://45uevg34gwlltnbsf2plyua1-wpengine.netdna-ssl.com/wp-content/uploads/2019/07/OXIS-Li-S-Ultra-Light-Cell-spec-sheet-v4.2.pdf
Zenlabs 12 Ah	NMC	SiOx	Pouch	12.3	42.558	133	145	64	6.7	https://www.energy.gov/sites/prod/files/2020/05/f75/bat247_lopez_2020_p_5.11.20_725PM_LR.pdf
Zenlabs 51 Ah	NMC	SiOx	Pouch	51	176.46	592	302	102	10	https://static1.squarespace.com/static/59dbcd906f4ca35190c9aeb4/t/5cac327223d25a000188813b/1554788979381/EPS+Range.pdf
SVOLT NMx 115 Ah	NMx	Gr	Prismatic	115	430	1760	220	102.5	33.4	https://insideevs.com/news/483181/svolt-cobalt-free-nmx-cell-available-order/
Cuberg HP-5P	NMC	Li	Pouch	5.1	19.635	52.9	80	60	6.2	https://www.datocms-assets.com/40252/1614987038-doeresultsforcuberg.pdf
Cuberg HV	NMC	Li	Pouch	5.1	19.4	48.1	77	59	5.3	https://s3.us-west-2.amazonaws.com/secure.notion-static.com/e6ea326b-48ae-4b47-a0ed-0f34a6884ec1/Screen_Shot_2022-01-11_at_11.26.05.png?X-Amz-Algorithm=AWS4-HMAC-SHA256&X-Amz-Content-Sha256=UNSIGNED-PAYLOAD&X-Amz-Credential=AKIAT73L2G45EIPT3X45%2F20220111%2Fus-west-2%2Fs3%2Faws4_request&X-Amz-Date=20220111T110149Z&X-Amz-Expires=86400&X-Amz-Signature=d87d4aa8142f9fffeeae8d6ba285cea081bf79afaa07bf3521bec8ff8e5426f2&X-Amz-SignedHeaders=host&response-content-disposition=filename%20%3D%22Screen%2520Shot%25202022-01-11%2520at%252011.26.05.png%22&x-id=GetObject
Sion Power Licerion	NMC	Li	Pouch	20	76.4	154	100	100	10	https://sionpower.com/products/
Enovix EX1-341729A	NMC	Si	Pouch	0.337	1.22	4.71	28.72	17.34	3.39	https://assets.website-files.com/6023ee57b22bf2f0c312206d/602d3e2d1b41897a1bb7c1c1_Enovix Wearable Cell Data Sheets.pdf
Prologium PLCB_36D3L8AAJA	NMC	Li	Pouch	8.3	31.125	115.28	133	218	3.7	https://docplayer.net/95626946-Prologium-lithium-ceramic-battery-profile.html
Solid Energy Hermes	NMC	Li	Pouch	3.4	13	29	66	37	6.35	http://assets.solidenergysystems.com/wp-content/uploads/2017/09/08171937/Hermes_Spec_Sheet1.pdf
LG E61V	NMC	Gr	Pouch	61.1	224.7	874.7	355	110	11.39	https://www.batemo.de/products/batemo-cell-library/e61v/
CALB LI48N50B	NMC	Gr	Prismatic	51.3	191.9	855.7	148	97.5	26.5	https://www.batemo.de/products/batemo-cell-library/l148n50b/
Samsung BMW530e	NMC	Gr	Prismatic	29.5	109.4	702	173	85	22	https://www.batemo.de/products/batemo-cell-library/bmw-530e/
SAPB VW eGolf	NMC	Gr	Prismatic	39.9	148	802.6	148	90	26	https://www.batemo.de/products/batemo-cell-library/vw-egolf/

Panasonic NCR2170-M	NCA	Gr-Si	Cylindrical	4.98	18.1	66.9	20.9	70.02		https://www.batemo.de/products/batemo-cell-library/ncr2170-m/
Panasonic NCR18650-G	NCA	Gr-Si	Cylindrical	3.34	12.3	46.4	18.25	65.1		https://www.batemo.de/products/batemo-cell-library/ncr18650-g/
AMTE Ultra Safe	NaMO2	Hard carbon	Pouch	30	93	655	228	167	11.6	https://amtepower.com/wp-content/uploads/2020/05/ULTRA-Safe-AMTE-A5-leaflet.pdf
HiNa NaCR26650	NaMO2	Soft carbon	Cylindrical	2.3	7.36	50.1	26	65		http://cpl.iphy.ac.cn/Y2021/V38/I7/076501#1
Toshiba SCiB High Power 2.9 Ah	NMC	LTO	Prismatic	2.9	6.96	150	63	97	14	https://www.global.toshiba/ww/products-solutions/battery/scib/product/cell.html
Toshiba SCiB High Power 10 Ah	NMC	LTO	Prismatic	10	24	510	116	106	22	https://www.global.toshiba/ww/products-solutions/battery/scib/product/cell.html
Toshiba SCiB High Energy 20 Ah	NMC	LTO	Prismatic	20	46	515	116	106	22	https://www.global.toshiba/ww/products-solutions/battery/scib/product/cell.html
Toshiba SCiB High Energy 23 Ah	NMC	LTO	Prismatic	23	52.9	550	116	106	22	https://www.global.toshiba/ww/products-solutions/battery/scib/product/cell.html
Sion Power Li-S	Sulfur	Li	Prismatic	2.8	5.805	16	37	55	11	https://iba2013.icmab.es/images/files/Wednesday/Morning/Yuriy Mikhaylik.pdf
iPhone X	LCO	Gr	Prismatic	2.716	10.34796	40	34	123.4	4	https://www.nasa.gov/sites/default/files/atoms/files/beyond_li-ion_battery_high_energy_and_power_cells_market_for_conferences.pdf
Samsung 3 Ah pouch	LCO	Gr	Pouch	3	11.55	42.7	77	40	5	https://www.nasa.gov/sites/default/files/atoms/files/beyond_li-ion_battery_high_energy_and_power_cells_market_for_conferences.pdf
Smartphone 3.5 Ah pouch	LCO	Gr	Pouch	3.5	13.48	51.3	80	45	5	https://www.nasa.gov/sites/default/files/atoms/files/beyond_li-ion_battery_high_energy_and_power_cells_market_for_conferences.pdf
Amprius HAPS	NMC	Si	Pouch	3.8	13.9	33.1	50	55	4.5	https://www1.grc.nasa.gov/wp-content/uploads/5.-Amprius.pdf
Amprius Drones HP	NMC	Si	Pouch	2.8	10.1	27.8	50	55	4.2	https://www1.grc.nasa.gov/wp-content/uploads/5.-Amprius.pdf
Amprius Drones LE	NMC	Si	Pouch	3.6	13.2	31.7	50	55	4.6	https://www1.grc.nasa.gov/wp-content/uploads/5.-Amprius.pdf
Amprius High Capacity	NMC	Si	Pouch	8.1	29.3	68.1	105	50	4.5	https://www1.grc.nasa.gov/wp-content/uploads/5.-Amprius.pdf
Amprius Army Wearable	NMC	Si	Pouch	5.4	21	49.5	54	64	5.4	https://www1.grc.nasa.gov/wp-content/uploads/5.-Amprius.pdf
KeepPower ICR18650-300PCM-R Protected	LCO	Gr	Cylindrical	3	11.1	48	19.3	69.5		https://www.tme.eu/Document/dcf728d9ce7d6edbd8bfbdd0321153d7/ACCU-18650-15APCB.pdf
Samsung ICR18650-29E	LCO	Gr	Cylindrical	2.9	10.73	44.5	18.4	65		https://batterybuilding.en.made-in-china.com/product/VvwQnMTHgIkR/China-Icr18650-29e-3-7V-2900mAh-18650-Lithium-Battery.html

Samsung ICR18650-26J	LCO	Gr	Cylindrical	2.6	9.438	45	18.4	65		https://datasheetspdf.com/pdf-file/1402663/Samsung/ICR18650-26J/1
Molicel IMR-18650E	LMO	Gr	Cylindrical	1.4	5.32	42	18.24	65		https://datasheetspdf.com/pdf/821868/E-ONEMOLIENERGY/IMR-18650E/1
Nitecore IMR18650 3100 mAh	LMO	Gr	Cylindrical	3.1	11.47	45.8	18.4	65		https://www.batteryjunction.com/nitecore-imr18650-3100-35a-battery.html
Nitecore IMR18650 2600 mAh	LMO	Gr	Cylindrical	2.6	9.62	44.1	18.2	65.2		https://files.batteryjunction.com/frontend/files/nitecore/msds/NITECORE-IMR18650-2600-40-MSDS.pdf
Nitecore IMR18650 2100 mAh	LMO	Gr	Cylindrical	2.1	7.77	42.7	18.3	65.1		https://sep.yimg.com/ty/cdn/theshorelinemarket/NITECORE-IMR18650-2100-30-MSDS.pdf?t=1643083619&
Panasonic NCR18650	NCA	Gr	Cylindrical	2.75	9.9825	46.5	18.5	65.3		https://www.omnitron.cz/_dokumenty/792019122953866/ncr-18650.pdf

Supplementary Note 1: Calculations of theoretical specific energy and energy density

We here consider two cases, $LiNi_{0.9}Co_{0.05}Al_{0.05}O_2$ (NCA) vs a graphite-silicon composite in small cylindrical cell format (approximately corresponding to concepts used by companies such as Tesla), and LFP/Gr in CTP concept (as demonstrated by companies such as BYD and CATL).

Theoretical maximum specific energy and energy density

For the NCA/Gr-SiOx, we assume that the anode is 96.5% wt% Gr (C_6) and 3.5% Si wt% active material, and only graphite for the LFP case. We use the following relative molecular masses:

M_r (NCA) = 96.05 g mol^{-1}

M_r (LFP) = 157.8 g mol^{-1}

M_r (C_6) = 72.066 g mol^{-1}

Mr (Si) = 28.086 g mol^{-1}

Using the formula $Q = \frac{1}{3.6} \cdot \frac{nF}{M_r}$ we obtain the following theoretical capacities in Ah kg^{-1}, where for Si, n = 15/4 (assuming final stoichiometry of $Li_{15}Si_4$) and n = 1 for all others:

Q_{NCA} = 279.1 Ah kg^{-1}

Q_{LFP} = 169.8 Ah kg^{-1}

Q_{Gr} = 371.9 Ah kg^{-1}

Q_{Si} = 3578.5 Ah kg^{-1}

The theoretical specific capacity on materials level for a pair of electrodes can be calculated for these two cases as:

$$\frac{1}{Q_{total}} = \frac{1}{Q_{cathode}} + \frac{1}{Q_{anode}}$$

For the NCA/Gr-SiOx case we have:

$$Q_{NCA,GrSiOx} = \frac{1}{\frac{1}{279.1} + \frac{1}{0.965 \cdot 371.9 + 0.035 \cdot 3578.5}} = 177.0\, Ah\, kg^{-1}$$

and for LFP:

$$Q_{LFP,Gr} = \frac{1}{\frac{1}{169.8} + \frac{1}{371.9}} = 116.6\, Ah\, kg^{-1}$$

To calculate energy density we can multiply the energy density by the nominal voltage. For NCA, in practice only 70-75% of the theoretical capacity is accessible in the potential range 3.5 – 4.2 V vs Li/Li^+, with a nominal voltage of ~3.6-~3.7 V for the full cell. If we assume a linear extrapolation of voltage up to the theoretical capacity of the material, we can assume for this case that the maximum nominal cell voltage where all of the NCA capacity could be accessed could lie in the range 3.9-4.0 V.

On this basis the theoretical energy density can therefore be estimated as:

$$W_{NCA/GrSiOx} = Q_{NCA/GrSiOx} \cdot 3.95 \pm 0.05 = 699 \pm 9 \; Wh \; kg^{-1}$$

and for LFP the cell voltage is constant and approx. 3.2 V:

$$W_{LFP/Gr} = Q_{LFP/GrSiOx} \cdot 3.2 = 373 \; Wh \; kg^{-1}$$

To calculate volumetric energies we can assume the following bulk densities of the materials:

ρ_{NCA} = 4.85 kg L^{-1}

ρ_{LFP} = 3.45 kg L^{-1}

ρ_{Gr} = 2.24 kg L^{-1}

ρ_{Si} = 2.33 kg L^{-1}

We can calculate the volumetric capacity for each material by multiplying the specific capacity by the density:

$$Q_{vol,NCA/GrSiOx} = \frac{1}{\frac{1}{279.1 * 4.85} + \frac{1}{(0.965 * 371.9 * 2.24) + (0.035 * 3579.1 * 2.33)}} = 605.6 \; Ah \; L^{-1}$$

Dividing this by the specific capacity obtained earlier (177 Ah kg^{-1}) gives an average density for the active materials (in capacity-balanced quantities) of 3.42 kg L^{-1}. For LFP the corresponding charge density is:

$$Q_{vol,LFP/Gr} = \frac{1}{\frac{1}{169.8 * 3.45} + \frac{1}{371.9 * 2.24}} = 343.9 \; Ah \; L^{-1}$$

which similarly gives an average material density of 2.95 kg L^{-1}.

Multiplying these densities by the theoretical specific energies obtained previously we arrive at theoretical energy densities of 2391 ± 31 Wh L^{-1} for NCA/Gr-SiOx and 1100 Wh L^{-1} for LFP/Gr.

Reversible capacities on materials level

We can assume the following *reversible* capacities (i.e., the specific charge which can in practice be reversibly accessed over multiple charge/discharge cycles) for the following materials:

NCA: ~200 mAh g^{-1}

Graphite: ~350 mAh g^{-1}

Silicon: ~2000 mAh g^{-1}

LFP: ~160 mAh g^{-1}

Assuming n/p =1, the total specific capacity on materials level can be calculated for these two cases as:

$$\frac{1}{Q_{total}} = \frac{1}{Q_{cathode}} + \frac{1}{Q_{anode}}$$

So for NCA:

$$Q_{NCA,GrSiOx} = \frac{1}{\frac{1}{200} + \frac{1}{0.965 \cdot 350 + 0.035 \cdot 2000}} = 134.2\ mAh\ g^{-1}$$

and for LFP:

$$Q_{LFP,Gr} = \frac{1}{\frac{1}{160} + \frac{1}{350}} = 109.8\ mAh\ g^{-1}$$

Taking typical cell voltages of 3.63 V and 3.2 V for NCA and LFP cells respectively, we arrive at "reversible" capacities on the materials level of 530 Wh kg^{-1} for NCA/Gr-SiOx and 351 Wh kg^{-1} for LFP/Gr. Following the previous calculations to determine a reversible volumetric capacity:

$$Q_{vol,NCA/GrSiOx} = \frac{1}{\frac{1}{200 * 4.85} + \frac{1}{(0.965 * 350 * 2.24) + (0.035 * 200 * 2.33)}} = 472.1\ Ah\ L^{-1}$$

$$Q_{vol,LFP/Gr} = \frac{1}{\frac{1}{160 * 3.45} + \frac{1}{350 * 2.24}} = 323.9\ Ah\ L^{-1}$$

From which we similarly arrive at effective densities of 3.52 kg L^{-1} and 2.95 kg L^{-1} for NCA/Gr-SiOx and LFP/Gr respectively, and subsequently 1866 Wh L^{-1} and 1035 Wh L^{-1} for LFP/Gr.

We consider the following ranges of cell level and pack level energy densities reasonable for each concept, on the basis of commercially available products:

NCA/GrSiOx, small cylindrical: 245-270 Wh kg^{-1}, 650-700 Wh L^{-1} (cell level); 155-175 Wh kg^{-1}, 245-290 Wh L^{-1} (pack level)

LFP/Gr, cell-to-pack: 160-200 Wh kg^{-1}, 350-400 Wh L^{-1} (cell level); 130-150 Wh kg^{-1}, 210-240 Wh L^{-1} (pack level)

We also assume that a typical usable state-of-charge (SoC) range for these systems is 5-90% for NCA/Gr-SiOx (i.e., 85% of total) and 5-100% for LFP/Gr (i.e., 95% of total).
